# Persistent mycobacteria evade an antibacterial program mediated by phagolysosomal TLR7/8/MyD88 in human primary macrophages

**DOI:** 10.1371/journal.ppat.1006551

**Published:** 2017-08-14

**Authors:** Alexandre Gidon, Signe Elisabeth Åsberg, Claire Louet, Liv Ryan, Markus Haug, Trude Helen Flo

**Affiliations:** 1 Centre of Molecular Inflammation Research and Department of Cancer Research and Molecular Medicine, Faculty of Medicine, NTNU, Norwegian University of Science and Technology, Trondheim, Norway; 2 The Central Norway Regional Health Authority, Trondheim, Norway; McGill UniversityHealth Centre, CANADA

## Abstract

Pathogenic mycobacteria reside in macrophages where they avoid lysosomal targeting and degradation through poorly understood mechanisms proposed to involve arrest of phagosomal maturation at an early endosomal stage. A clear understanding of how this relates to host defenses elicited from various intracellular compartments is also missing and can only be studied using techniques allowing single cell and subcellular analyses. Using confocal imaging of human primary macrophages infected with *Mycobacterium avium* (Mav) we show evidence that Mav phagosomes are not arrested at an early endosomal stage, but mature to a (LAMP1^+^/LAMP2^+^/CD63^+^) late endosomal/phagolysosomal stage where inflammatory signaling and Mav growth restriction is initiated through a mechanism involving Toll-like receptors (TLR) 7 and 8, the adaptor MyD88 and transcription factors NF-κB and IRF-1. Furthermore, a fraction of the mycobacteria re-establish in a less hostile compartment (LAMP1^-^/LAMP2^-^/CD63^-^) where they not only evade destruction, but also recognition by TLRs, growth restriction and inflammatory host responses that could be detrimental for intracellular survival and establishment of chronic infections.

## Author summary

*Mycobacterium avium* is increasingly reported as a causative agent of non-tuberculous disease in immunocompromised patients and in individuals with underlying disease or using immunosuppressant drugs, with prevalence often higher than the more pathogenic *M*. *tuberculosis* in developed countries. Both *M*. *avium* and *M*. *tuberculosis* cause persistent infections by surviving inside host macrophages. Here, we identify from which compartment *M*. *avium* evoke inflammatory signaling in human primary macrophages, and the pattern-recognition receptors involved. In essence, we present three key findings: 1) *M*. *avium* phagosomes are not arrested at an early endosomal stage, but rather mature normally into phagolysosomes from where a fraction of the bacteria escape and re-establish in a new compartment. 2) In addition to avoiding degradation in phagolysosomes, by escaping *M*. *avium* also evade inflammatory signaling. 3) *M*. *avium* unable to escape is degraded in phagolysosomes and recognized by Toll-like receptors 7 and 8. Our results can contribute to new understanding of intracellular infections, and thus have vital clinical implications for development of novel anti-microbial strategies and host-targeted therapy to mycobacterial and other infectious diseases.

## Introduction

Both *Mycobacterium tuberculosis* (Mtb) and pathogenic non-tuberculous mycobacteria like *M*. *avium* (Mav) have developed mechanisms to hijack the normal trafficking of phagosomes and use macrophages as a natural habitat and tools of spread in the host [1–3]. However, the spatiotemporal dynamics of how intracellular trafficking of mycobacteria relates to recognition by pattern recognition receptors (PRRs) and elicitation of antibacterial responses by host macrophages is not fully understood, in particular for Mav. Mav can establish chronic infections and clinical disease that is hard to treat such as pulmonary disease, lymphadenitis or disseminated infection [4,5]. In search of new therapeutic strategies to shorten the treatment of mycobacterial diseases and meet the increasing drug resistance it is thus crucial to understand the minimum infectious unit, the mycobacterium-infected macrophage.

Macrophages initiate different destruction programs depending on the particular intruder encountered. The process begins with the engagement of pathogen-recognition receptors (PRR) like Toll-like receptors (TLRs) and C-type lectins at the plasma membrane, initiating inflammatory signaling followed by phagocytosis and the gradual maturation of the phagosome into a phagolysosome, where bacteria are attempted digested [6]. Different mycobacterial ligands are displayed and activate distinct PRRs present in the various cellular compartments [6,7]. TLR2/1/6 are expressed on the plasma membrane where they recognize mycobacterial lipoproteins, proteins and glycolipids [8,9]. TLR3, 7, 8 and 9 depend on the protein Unc-93 homolog B1 (UNC93B1) to traffic from the endoplasmic reticulum to endolysosomal compartments where they detect nucleic acids [10,11]. TLR9 has previously been shown to recognize mycobacterial DNA [12,13]. TLR7 and TLR8 recognize single-stranded RNA and RNA degradation products from viruses and bacteria, and polymorphisms in TLR7 and 8 have been associated with increased susceptibility to pulmonary tuberculosis [11,14–22]. However, whether TLR7/8 play a role in recognizing mycobacteria is unclear. When activated, TLR-ligand complexes are bridged by sorting adaptors like toll-interleukin 1 receptor (TIR) domain-containing adaptor protein (TIRAP) or TRIF-related adaptor molecule (TRAM) to signaling adapter molecules such as Myeloid differentiation primary response gene 88 (MyD88) and TIR-domain-containing adapter-inducing interferon-β (TRIF) in raft domains of various sub-cellular membranes. Signaling cascades culminate in the nuclear translocation of transcription factors like nuclear factor (NF)-κB and interferon regulatory factors (IRFs) and subsequent production of inflammatory mediators, type I interferons (IFNs) and antimicrobial programs [6,23,24].

The innate inflammatory response is central in anti-mycobacterial host defenses. Mice deficient in MyD88 are susceptible to infections with either Mtb or Mav [25,26], and we have recently demonstrated that negative regulation of inflammatory signaling by a cellular stress sensor, Keap1, facilitates intracellular growth of Mav in human primary macrophages [27]. However, mycobacteria are intracellular pathogens that are capable of evading most antimicrobial strategies and killing through poorly characterized mechanisms that involve arrest of phagosomal maturation and lysosomal delivery, while access to essential nutrients is retained [1,2]. Phagosomal maturation arrest is often suggested to happen at an early endosomal stage for several reasons: Mtb and Mav maintain the pH of their compartment at 6–6.5 by excluding vATPase and preventing fusion with lysosomes [28–30]. The mycobacterial compartments also stain positive for transferrin receptor and retain the early endosomal marker Rab5, but not the late endosomal marker Rab7, although Rab7 was shown by Koide's group to be transiently present on Mtb phagosomes [31–33]). We have previously shown that the Mav compartment (MavC) interacts with the Rab11^+^ recycling endocytic pathway, from where Mav can access transferrin-iron and avoid the antibacterial protein lipocalin 2 [34,35].

To dissect from which compartment(s) Mav elicits inflammatory signaling, the mechanism(s) involved and how these relate to phagosomal maturation arrest and mycobacterial survival, we investigated the spatiotemporal recognition of Mav infection at a sub-cellular level in human primary macrophages using confocal microscopy. We found that Mav-containing phagosomes mature to LAMP1^+^ late endosomes/phagolysosomes (MavPLs) where growth is prevented and inflammatory signaling is activated through TLR7/8. Furthermore, we found that a fraction of Mav re-established in a less hostile environment, the LAMP1^-^ MavCs, thereby evading TLR activation and subsequent inflammatory signaling, cytokine production and growth restriction. Thus, the MavC represents a safe house for Mav, shielding it both from lysosomal destruction and detection by TLRs.

## Results

### *Mycobacterium avium* phagosome maturation proceeds via EEA1^+^ early endosomes and LAMP1^+^ phagolysosomes to LAMP1^-^ compartments (MavCs) that support growth

**Fig 1:**
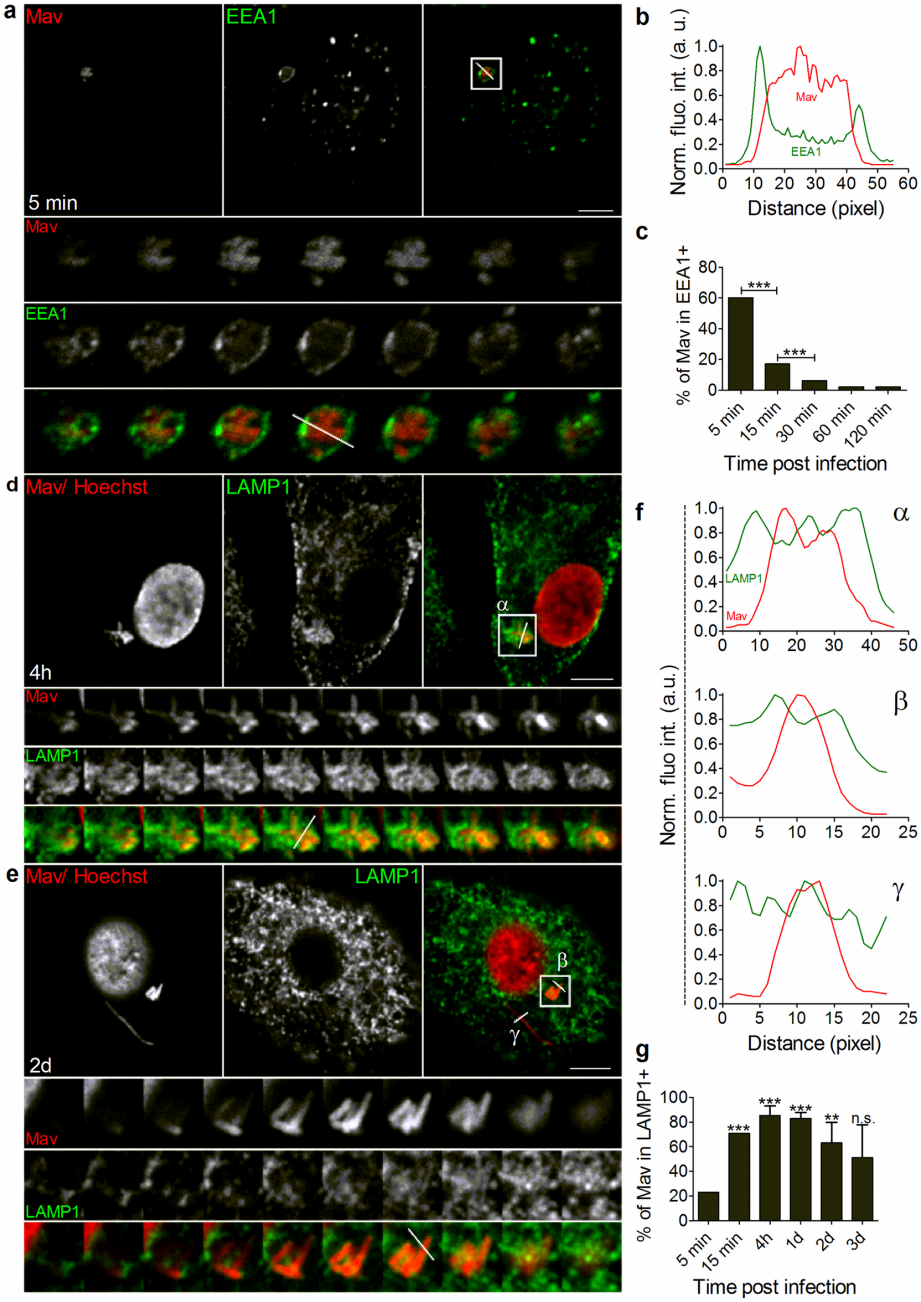
Mav temporarily resides in the phagolysosomal compartment. Human MDMs were infected with Mav-CFP (red) for 10 min followed by a chase for 5 minutes to 3 days, stained for EEA1 ((A), early endosomes, green) or LAMP1 ((D, E) late endosomes/lysosomes, green) using antibodies and for nuclei using Hoechst (red), and analyzed by confocal microscopy at the indicated time points. Single labeling (left and middle images) and merged images (right images) are shown. Bottom-to-top projections of 3D-stacks from boxed areas are shown in lower panels (left to right) and represent Mav, EEA1/LAMP1^+^ membranes and merged images. Quantification of Mav localization in EEA1^+^ or LAMP1^+^ compartments was performed on 3D stacks using fluorescence intensity profiles along the indicated line (*FAL*, (B) and (F), Mav: red line; EEA1/LAMP1: green line). For each time point, at least 70 cells were recorded per donor. Quantification graphs represent mean value +/- SEM of Mav localization in EEA1^+^ (C) or LAMP1^+^ (G) compartments for 3 donors. *P* values were calculated using Fisher Exact Test (* *P* <0.05, ** *P* <0.01 and *** *P* <0.005). a.u.: arbitrary unit. Scale bar represents 10 μm.

It is well known that mycobacterial pathogens escape killing by host macrophages and reside in a compartment where they can access nutrition [7,30,34,36]. However, most studies rely on few time points of infection with incomplete and often incongruent results regarding the spatiotemporal location/movement of mycobacteria inside macrophages, leaving the exact trafficking pathways to be elucidated. We infected human monocyte-derived macrophages (MDMs) with Mav expressing CFP (Mav-CFP) for 10 minutes (pulse) and chased for several time points between 5 minutes and 3 days. Confocal microscopy revealed that bacteria are first internalized in early endosomes, as shown by early endosome antigen 1 (EEA1) immuno-labeling (Fig 1A, bottom-to-top projection of 3D-stack from boxed area is shown in lower panels). Profiling the fluorescence intensity along the line (*FAL*) of Mav phagosomes confirmed that mycobacteria were enclosed by EEA1^+^ membranes (Fig 1B). Quantification over time showed a significant decrease of EEA1 associated with Mav phagosomes already 5 to 15 minutes after uptake (*P* <0.005), indicative of rapid maturation of Mav phagosomes that remained EEA1 negative from 30–60 minutes onwards (Fig 1C).

Pathogenic mycobacteria were previously thought to arrest phagosomal maturation at an early endosomal stage based on the lack of Rab7 recruitment or retainment [31–33], v-ATPase exclusion and lack of acidification [28]. Mycobacterial phagosomes are however, heterogeneously harboring markers of late endosomes/lysosomes like lysosomal-associated membrane protein 1 (LAMP1), LAMP2 and LAMP3/CD63 [28,29,37,38]. Correspondingly, we found that Mav phagosomes temporarily acquired LAMP1^+^ staining (Fig 1D and 1E, bottom-to-top projection of 3D-stack from boxed area is shown in lower panels, and corresponding *FAL* profiling in Fig 1F). Quantification over time showed that 70% of Mav resided in LAMP1^+^ late endosomes/phagolysosomes as early as 15 minutes post infection, increasing to more than 90% 4 hours to 1 day post infection, suggesting Mav phagosomes are not arrested at an early endosomal stage but mature normally to a late endosomal/lysosomal stage (Fig 1G, *P* <0.005 for both time points). Interestingly, 2–3 days post infection there was a significant decrease of Mav phagosomes associated with LAMP1 (Fig 1G), further suggesting sorting and escape of an increasing fraction of the mycobacteria from phagolysosomes (Fig 1E with *FAL* profiling in Fγ and quantifications over time in G). We confirmed the results by staining for LAMP2 and for CD63/LAMP3, two additional established markers of late endosomes/lysosomes, in combination with LAMP1 (S1 Fig). Mav stays compartmental as it lacks the ESX-1 secretion system suggested to facilitate translocation of Mtb and *M*. *marinum* to the cell cytosol [39–41]. These observations are also consistent with our previous findings that Mav is associated with transferrin and Rab11a after 4 days of infection [34].

**Fig 2:**
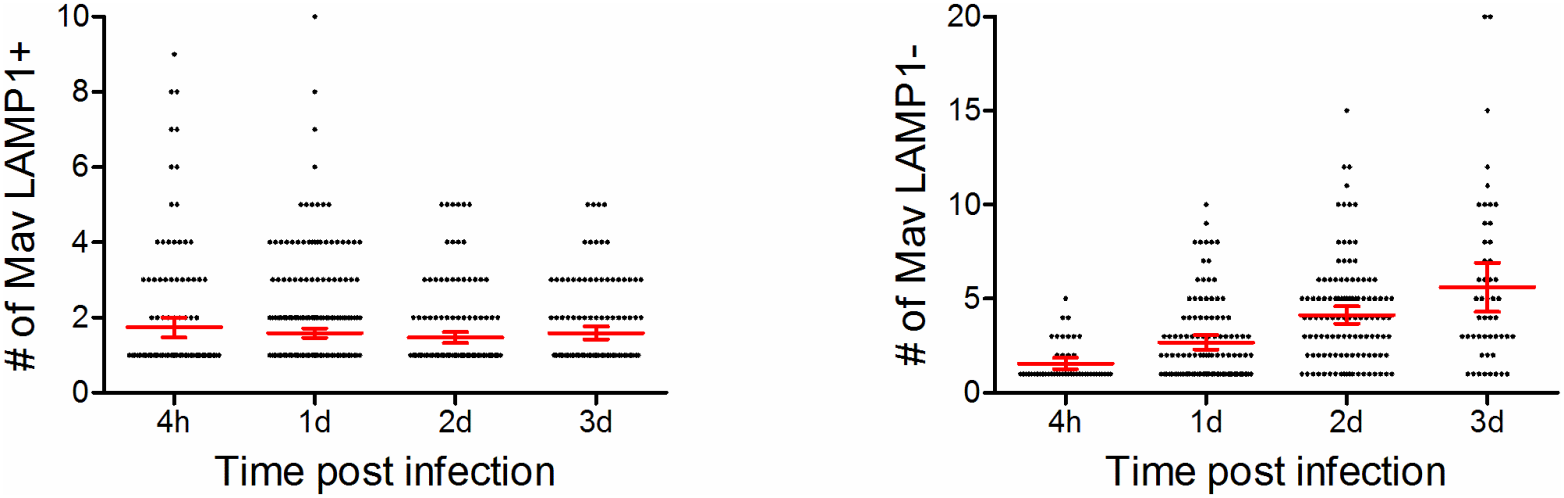
MavCs, but not MavPLs, support Mav replication. Human MDMs were infected with Mav-CFP for 10 min, chased for 5 min to 3d, fixed with 4% PFA and analyzed by confocal microscopy. *In situ* quantification of the actual number of Mav inside (left panel) or outside (right panel) LAMP1^+^ compartments at different time points, including the mean value +/- 95% confidence intervals.

Mycobacteria in general and Mav in particular are known to survive acidic conditions but may not replicate in fully matured phagolysosomes [1,42,43]. To assess the acidity of Mav phagosomes we did live-cell imaging of Mav-infected macrophages loaded with Cresyl violet, a membrane-permeant fluorophore that localizes to lysosomes and acidic vacuoles [44]. The fraction of acidic Mav phagosomes increased 1 day and decreased 2 days post infection, following the pattern of LAMP staining (S2 Fig). We then asked whether MavPLs or MavCs would support replication of Mav by quantifying of the number of Mav in LAMP1^+^ and LAMP1^-^ compartments over time (Fig 2). The number of Mav in MavPLs did not change whereas the number of Mav in MavCs steadily increased over the 3 days of infection, clearly demonstrating that MavCs, but not MavPLs, support growth of Mav.

### Phagolysosomes retain PFA-killed Mav

**Fig 3:**
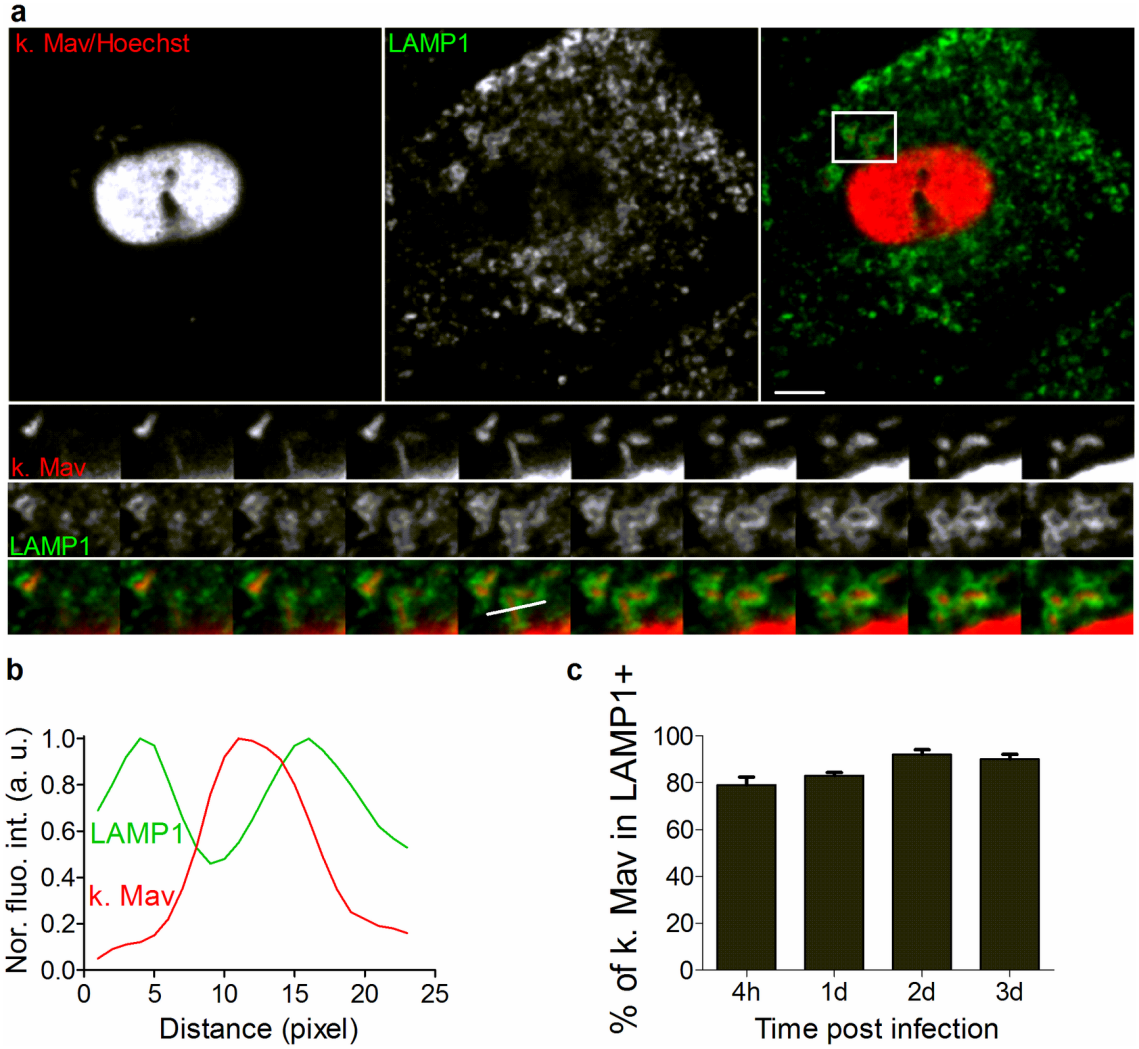
Phagolysosomes retain PFA-killed Mav. Human MDMs were exposed to PFA-killed Mav-CFP (10 min uptake, chased for 4h to 3d), stained with anti-LAMP1 antibody and Hoechst (nucleus) and analyzed using resolution-limited confocal microscopy. (A) LAMP1^+^ late endosomes/lysosomes (green), Mav and nuclei (red). Single labeling (left and middle images) and merged images (right images) are shown. Bottom-to-top projections of 3D-stack from boxed area are shown in lower panels and represent Mav, LAMP1^+^ membranes and merged images. Quantification of Mav localization in LAMP1^+^ compartments was performed on 3D stacks using fluorescence intensity profiling of Mav phagosomes along the indicated line ((B), Mav: red line; LAMP1: green line). For each time point, at least 70 cells were recorded for each donor. (**c**) The percentage of Mav localized to LAMP1^+^ compartments (mean value +/- SEM for 3 donors). *P* values were calculated using two-tailed t-test (* <0.05, ** <0.01 and *** <0.005.). a.u.: arbitrary unit. Scale bar represents 10 μm.

Infection with live Mav will over time yield a mixture of live and dead bacteria residing in different subcellular compartments, whereas dead Mav should all be routed to LAMP1^+^ phagolysosomes for degradation. To test this hypothesis we next assessed trafficking of paraformaldehyde (PFA)-killed Mav in human primary macrophages (Fig 3A–3C and S3 Fig). Phagosomes with dead Mav rapidly stained positive for LAMP1 as depicted in the bottom-to-top projection of a 3D-stack from the boxed area in Fig 3A and the corresponding *FAL* profiling (Fig 3B). Quantification over time demonstrated that, contrary to live Mav, PFA-killed Mav was retained in LAMP1^+^ phagolysosomes throughout the 3-day infection and significantly different from live Mav 3 days post infection (Figs 3C and 1G). We observed no difference in the uptake of live and PFA-killed Mav (S3B Fig). Our results thus suggest that live Mav temporarily reside in phagolysosomes before re-establishing in a new compartment allowing replication.

### Mav induces inflammatory signaling from LAMP1^+^ phagolysosomes in human primary macrophages

**Fig 4:**
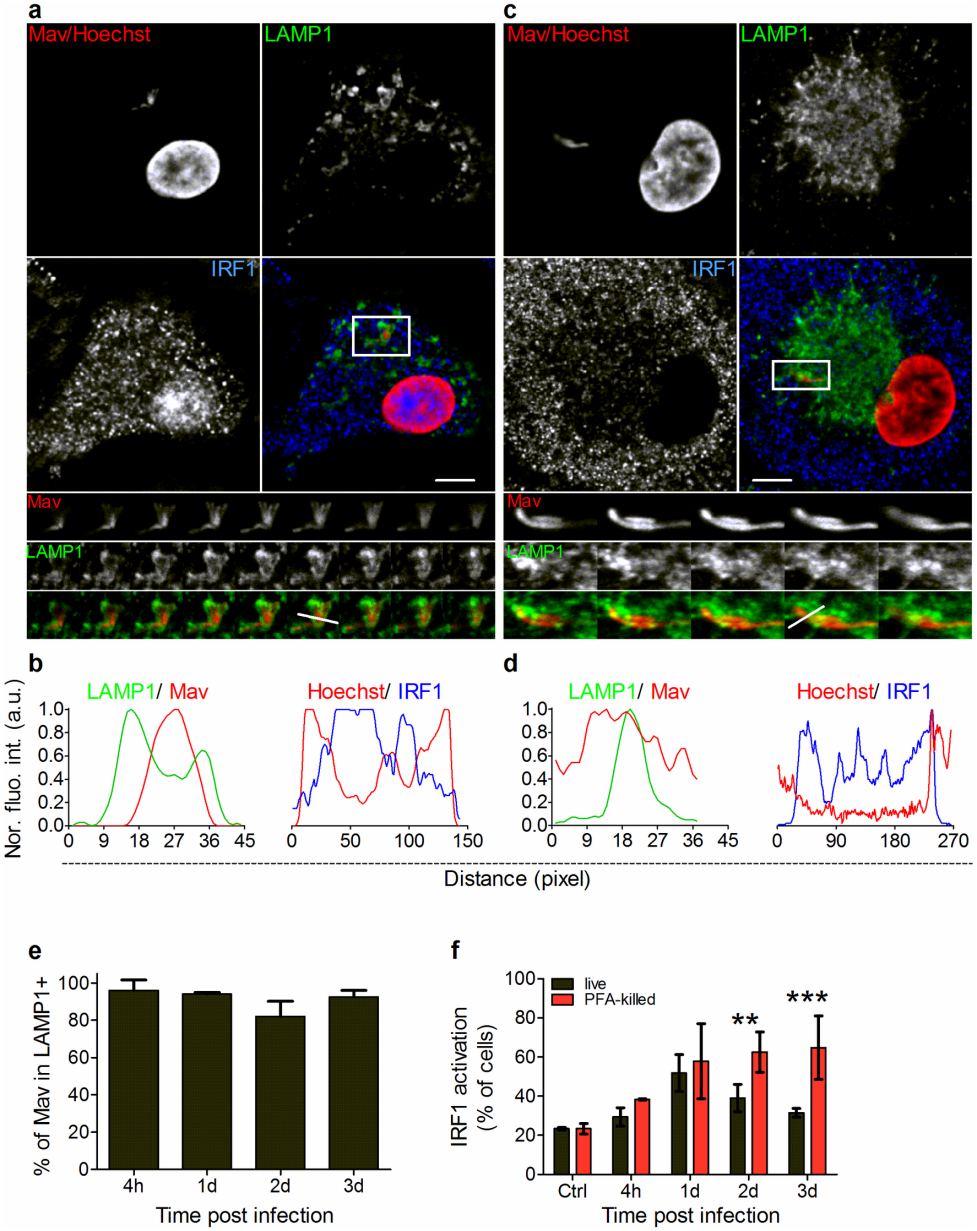
Activation of IRF-1 coincides with phagolysosomal localization of Mav. Human MDMs were infected with live or PFA-killed Mav-CFP (red) for 10 min, chased for 4h to 3d and stained with antibodies to IRF-1 (blue) and LAMP1 (green) before analysis of MDMs containing only single Mav using confocal microscopy. Nuclei were revealed by staining with Hoechst (red). Single and merged images are shown; bottom-to-top projections of 3D-stacks from boxed areas in lower panels represent Mav, LAMP1 and merged images (A, C). (A) Cell with live Mav in LAMP1^+^ compartment inducing nuclear translocation of IRF-1. (C) Cell with live Mav in LAMP1^-^ compartment and no nuclear translocation of IRF-1. (B, D) Quantification of live Mav localization in LAMP1^+^ compartments and nuclear localization of IRF-1 was performed on 3D stacks using fluorescence intensity profiles along the indicated lines of Mav phagosomes ((B) and (D), left (Mav: red trace; LAMP1: green trace) and right (Hoechst: red trace; IRF-1: green trace) graphs respectively). (E) The fraction of live Mav localized to LAMP1^+^ phagolysosomes in IRF-1-activated cells (mean percentage +/- SEM from 3 donors). For each time point, at least 70 cells were recorded for each donor. (F) IRF-1 nuclear translocation at indicated time points after challenge with live or PFA-killed Mav. Quantification graphs represent the mean value +/- SEM for each time point for live Mav (black bars) and PFA-killed Mav (red bars) (n>600 cells per time point and per donor, 3 donors). *P* values between live Mav and PFA-killed Mav were calculated using two-tailed t-test (* *P* <0.05, ** *P* <0.01 and *** *P* <0.005.). a.u.: arbitrary unit. Scale bar represents 10 μm.

Macrophage inflammatory responses to mycobacterial pathogens are well described [2,7,8], but it is largely unknown from which subcellular compartments mycobacteria are recognized and signaling originates. NF-κB is one of the key transcription factors driving inflammatory responses, and we and others have demonstrated that IRF-1 is activated in various infections including mycobacteria [27,45–47]. Thus, we first monitored the nuclear translocation of NF-κB (phospho-p65) and IRF-1 4 hours to 3 days post Mav infection of human primary macrophages using confocal microscopy. Both pathways were significantly activated by Mav with IRF-1 slightly delayed compared to NF-κB (S4 Fig). Even single cell analyses may not be sufficient to fully relate trafficking to inflammatory signaling if the cell is infected with more than one microbe. We thus conducted 3D confocal microscopy of macrophages harboring only single bacteria and assayed for nuclear translocation of IRF-1 and Mav association with LAMP1 (Fig 4A–4D). Every cell was first scored as positive or negative for nuclear translocation of IRF-1, whereafter the location of the mycobacterium was determined (LAMP1^+^ or LAMP1^-^). IRF-1 was chosen over NF-κB simply because the NF-κB Ab that works for immunofluorescence and nuclear translocation assays is not compatible with the protocol we need to use for LAMP1 staining, whereas the IRF-1 Ab is. More than 90% of the cells that were positive for nuclear IRF-1 harbored Mav associated with LAMP1^+^ membranes at all time points measured over the 3 days infection (Fig 4A, 4B and 4E). Conversely, non-activated cells defined by absent nuclear IRF-1 harbored Mav in compartments devoid of LAMP1 (Fig 4C and 4D), suggesting that Mav induces inflammatory signaling from LAMP1^+^ phagolysosomal compartments, and not from MavCs.

Since PFA-killed Mav was retained in LAMP1^+^ phagolysosomes (Fig 3) the inflammatory response in cells with only dead Mav should reflect signaling mainly from this compartment. We thus compared the temporal activation by live and PFA-killed Mav of IRF-1 nuclear translocation and cytokine secretion from macrophages (Fig 4F and S4D Fig). PFA-killed Mav induced nuclear translocation of IRF-1 and the response was sustained over the 3-day time course in comparison to infection with live Mav, where the percentage of Mav-infected cells with nuclear IRF-1 declined with time (Fig 4F, P<0.005). There was no difference in uptake of live and PFA-killed Mav (S3B Fig). The nuclear translocation assay quantifies the frequency of activated Mav-infected macrophages but does not necessarily inform about the nature or magnitude of the various responses that usually involve multiple transcription factors, e.g. for cytokine production. Multiplex ELISA on cell supernatants revealed that, despite donor variations, PFA-killed Mav induced increased levels of secreted TNF, IL-6, IL-10 and IP-10, but not IL-8, compared to live Mav (S4D Fig). Taken together, our results suggest that PFA-killed Mav remains in phagolysosomes and induces strong and sustained inflammatory signaling whereas the fraction of live Mav residing in phagolysosomes decreases over time, coinciding with reduced inflammatory responses induced by Mav incapable of escaping. This also strengthens the evidence that Mav induces inflammatory signaling from LAMP1^+^ phagolysosomes and not LAMP1^-^ MavCs in human primary macrophages.

### MyD88 is recruited to LAMP1^+^ Mav phagolysosomes and not to LAMP1^-^ MavCs

**Fig 5:**
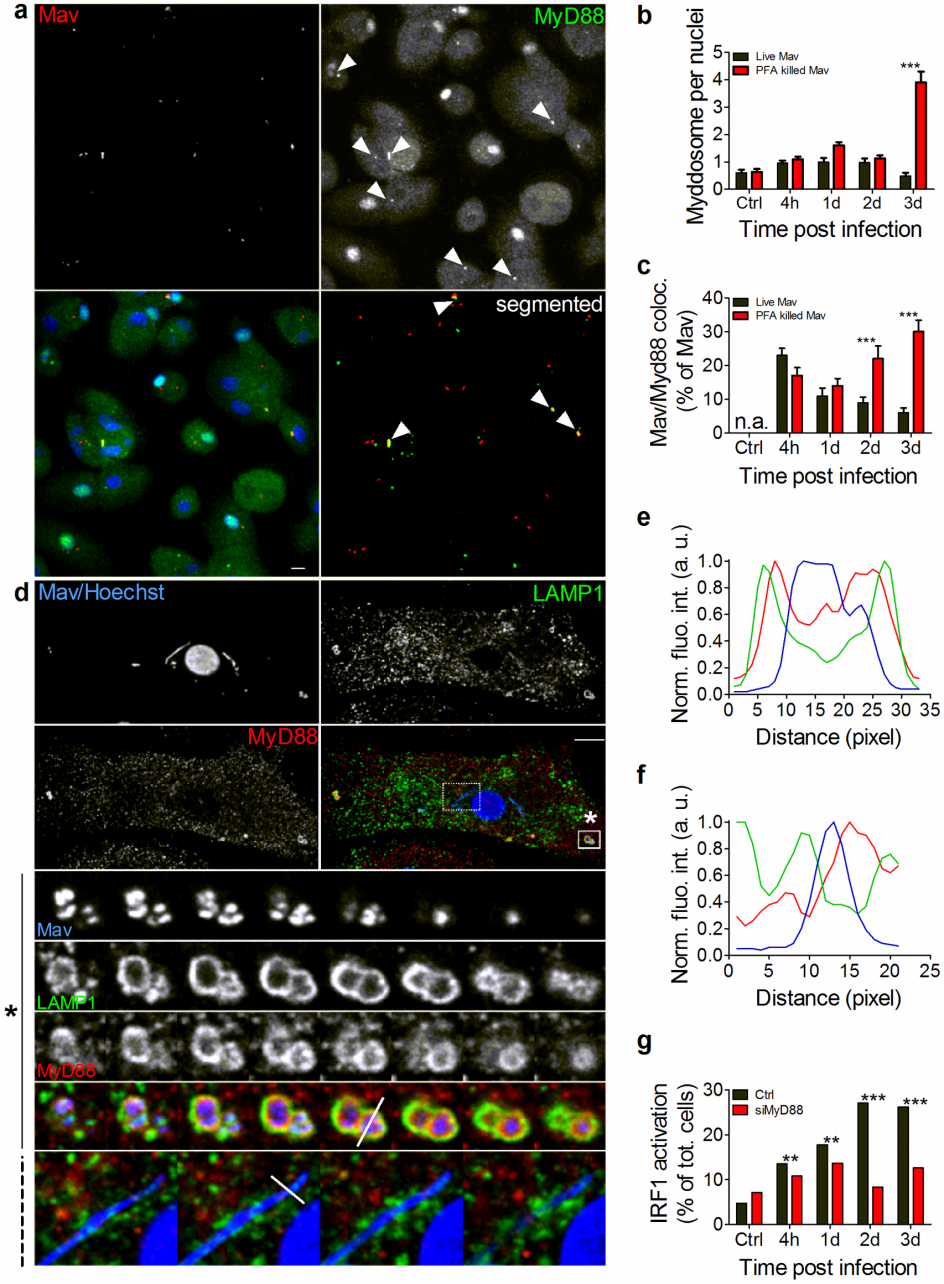
MyD88 is recruited to LAMP1^+^ Mav phagolysosomes and not to LAMP1^-^ MavCs. Human MDMs were challenged with live or PFA-killed Mav-CFP for 10 min, chased for 4h to 3d and stained with antibodies to MyD88, LAMP1 or with Hoechst (nuclei) and analyzed using non resolution-limited confocal microscopy. (A) Single stain, merged and segmented images are shown of Myddosome formation (MyD88 green) in MDMs infected with live Mav (red). (B-C) Quantification graphs represent the mean number +/- SEM of Myddosomes per nucleus (B, all cells counted) and MyD88-Mav association (C) at different time points after challenge with live Mav (black bars) or PFA-killed Mav (red bars) (n>300 cells per time point per donor, 3 donors). (D) Confocal microscopy imaging of Mav (blue) association with LAMP1^+^ compartments (green) and recruitment of MyD88 (red). Single and merged images are shown with bottom-to-top projections of 3D-stacks from boxed insets in lower panels: plain line box with star in periphery of the cell shows Mav enclosed in LAMP1^+^MyD88^+^ compartment (single and merged images from 3D stack); dashed line box next to nucleus shows Mav in LAMP1^-^MyD88^-^ compartment (merged images). (E) Quantification of Mav and MyD88 localization in LAMP1^+^ compartments was performed on 3D stacks using fluorescence intensity profiles along the indicated line (*FAL*) (Mav: blue trace; LAMP1: green trace; MyD88: red trace). (F) *FAL* of Mav compartment (MavC). (G) MDMs pre-treated with siRNA against MyD88 were infected with live Mav for 6 hours and stained for IRF-1 nuclear translocation before analysis using non resolution-limited confocal microscopy. Quantification graphs represent the mean value for each time point (n>700 cells per time point) of one representative donor from three independent experiments. *P* values between live Mav and PFA-killed Mav were calculated using two-tailed t-test for (B) and (C) and Fisher Exact test for (G) (* <0.05, ** <0.01 and *** <0.005). a.u.: arbitrary unit. Scale bar represents 10 μm.

To further validate the compartmental origin of Mav recognition and induction of host responses we next investigated the temporal activation and localization of the TLR signaling adaptor, MyD88. Immuno-staining of MyD88 in macrophages harboring live or PFA-killed Mav revealed the formation of aggregates (Myddosomes [48] as shown in Fig 5A). Quantification of Myddosomes by image segmentation based on particle size and fluorescence intensity displayed a pattern similar to IRF-1 activation: live Mav increased Myddosome formation over the first 24 hours followed by a decrease 2 and 3 days post infection, whereas the number of Myddosomes per cell increased substantially at day 3 in macrophages harboring PFA-killed Mav (Fig 5B). Co-localization analysis revealed that about 20% of Mav were associated with MyD88 4 hours post infection, decreasing over time for live Mav and, conversely, increasing over time for PFA-killed Mav (Fig 5C, *P* <0.005). Concomitant staining for LAMP1 revealed a specific recruitment of MyD88 to LAMP1^+^ Mav phagolysosomes as depicted in the bottom-to-top projection of the 3D-stack marked with an asterisk in Fig 5D (box with solid lines) and corresponding *FAL* profiling (Fig 5E).

Importantly, we were not able to detect MyD88 associated with LAMP1^-^ MavCs (Fig 5D, bottom-to-top projection of a 3D-stack from the dashed box, and corresponding *FAL* profiling (Fig 5F). Moreover, in LAMP1^+^MyD88^+^ phagolysosomes the morphology of Mav often appeared coccoid rather than rod-shaped, indicative of partial degradation, whereas mycobacteria residing in LAMP1^-^MyD88^-^ MavCs appeared intact and forming elongated chains with visible septa indicating growth and replication (Fig 5D). This is supported by results in Fig 2 showing that Mav numbers increased in MavCs over time, but not in MavPLs. Nuclear translocation of IRF-1 was markedly reduced over the first 3 days of infection in macrophages treated with siRNA to MyD88, suggesting MyD88 is conveying Mav-induced responses from the LAMP1^+^ phagolysosome (Fig 5G and S6 Fig). Our data thus suggest that Mav induces MyD88 recruitment and signaling only from LAMP1^+^ phagolysosomes, and by re-establishing in LAMP1^-^ compartments Mav not only escapes lysosomal destruction but also evades recognition and MyD88-mediated host responses.

### Mav is recognized by TLR7/TLR8 recruited to the phagolysosomal compartment

**Fig 6:**
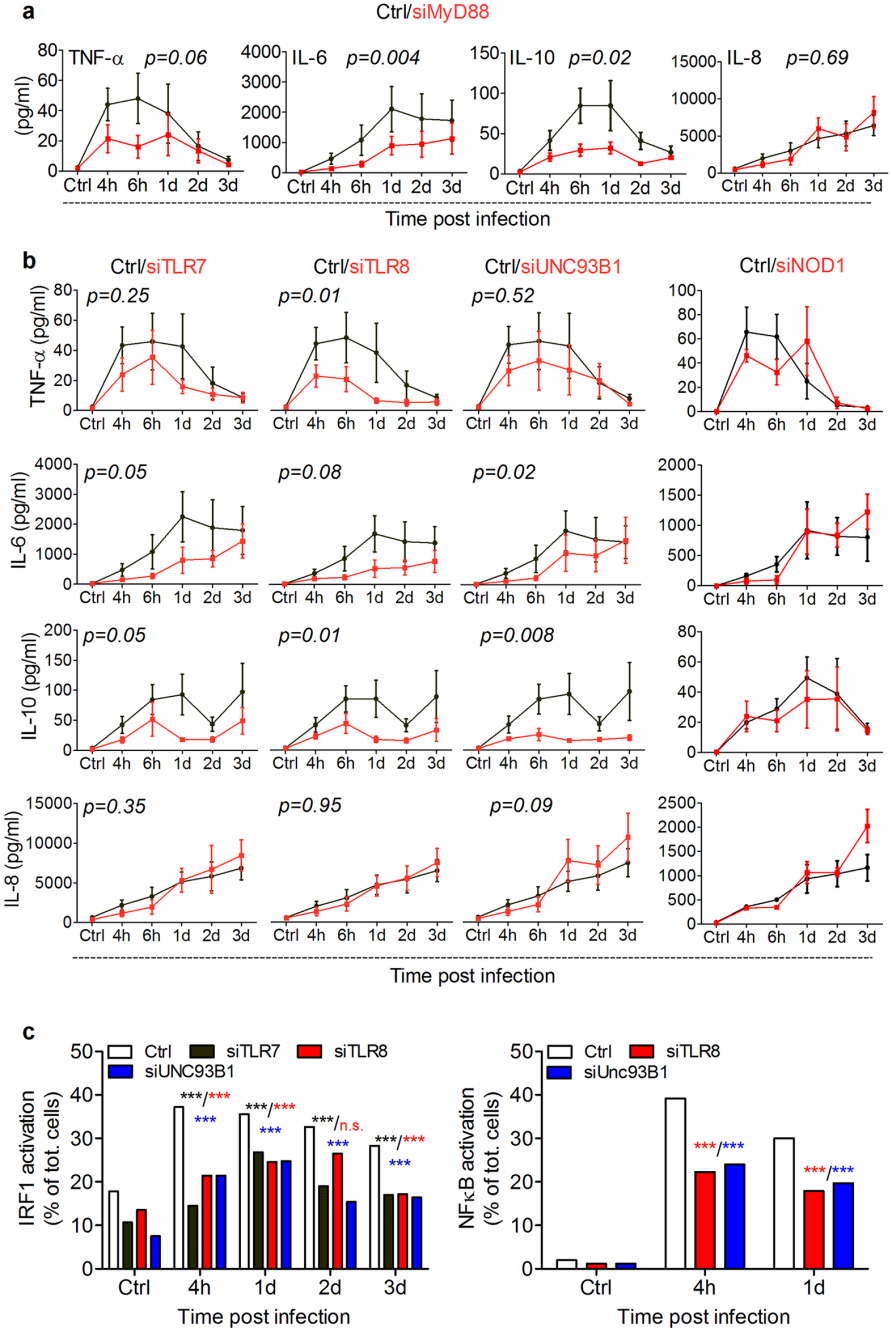
TLR7/8/MyD88 trigger inflammatory signaling induced by Mav infection. Human MDMs were treated with siRNA against target genes or a non-targeted control before infection with Mav-CFP (10 min uptake followed by chase for the indicated times). Cell supernatants were harvested at the indicated time points post infection and secreted cytokines assessed by multiplex ELISA. Cytokine responses from Mav-infected MDMs pretreated with siMyD88 (A), siTLR7, siTLR8, siUNC93B1 or siNOD1 (B). Graphs represent average concentrations +/- SEM of TNF-α, IL-6, IL-10 and IL-8 from at least 6 donors treated with target siRNA (red) or non-targeted siRNA (black). (C) Cells were pre-treated and infected as indicated, fixed and stained for IRF-1 and NF-κB nuclear translocation before analysis using non resolution-limited confocal microscopy. Quantification graphs represent the mean value for each time point (n>700 cells per time point) of one representative donor from three independent experiments. *P* values between Ctrl and each time point were calculated using Fisher Exact Test (* *P* <0.05, ** *P* <0.01 and *** *P* <0.005.).

To confirm a functional role for MyD88 in Mav-induced responses we next assessed secretion of inflammatory cytokines 4 hours to 3 days post infection of macrophages treated with siRNA to *MyD88* or non-targeted control (NTC) (Fig 6A, S6 and S7 Figs). Mav uptake was similar in siMyD88 and siNTC treated macrophages (S8A Fig). Despite variability between donors we found that TNF-α, IL-6 and IL-10 secretion was significantly decreased in siMyD88 treated cells (Fig 6A). We calculated the area under the curve (AUC) for each cytokine/donor couple to obtain an estimate of the total amount of secreted cytokines induced by the infection. Knocking down MyD88 considerably reduced the AUCs for TNF-α, IL-6 and IL-10 in 8/10, 7/10 and 8/10 of the donors, respectively, whereas IL-8 secretion was not affected (S7 Fig, cutoff set at 25% variation). The results further support that MyD88 is conveying Mav-induced responses from the phagolysosome, possibly initiated by Mav engaging endolysosomal TLRs.

Of the endolysosomal TLRs only TLR9 has been shown to respond to mycobacteria [21,49]. We also found TLR7 and 8 to be expressed in human primary macrophages (S6 Fig and [19]). Both TLR7 and 8 recognizes ssRNA in a species-specific manner [50] and we and others have shown that human TLR8 responds to bacterial RNA [11,15,19,20,51]. We next used siRNA to knock down TLR7, TLR8, and UNC93B1 in human primary macrophages, achieving an average reduction in mRNA levels of 80% (S6 and S7 Figs). UNC93B1 is needed for transport to and activity of TLR7/8 in endolysosomes [11]. Similar to what we observed with MyD88, knocking down TLR8 consistently and significantly reduced Mav-induced secretion of TNF-α, IL-6 and IL-10 by macrophages in 8/10, 7/10 and 8/10 of the donors (Fig 6B with accompanying AUCs in S7 Fig). A more variable reduction in cytokine levels was seen when knocking down TLR7 (6/9, 5/9, 7/9) or UNC93B1 (5/9, 6/9, 7/9), with IL-10 responses most consistently reduced. Interestingly, siTLR8 more consistently reduced cytokine responses compared to siTLR7 despite similar knockdown efficiencies, thus our data indicate non-redundancy between TLR7 and 8 in responding to Mav. As for MyD88, IL-8 responses were not affected by knocking down TLR7, 8 or UNC93B1 (Fig 6B and S7 Fig). A similar trend was also seen in response to PFA-killed Mav, with reduced cytokine responses when knocking down MyD88, TLR7/8 or UNC93B (S5 Fig). There was no difference in Mav uptake in target and control siRNA treated cells, indicating that none of these proteins are central for Mav phagocytosis (S8A Fig). Knockdown of the NOD1 or STING did not impact on Mav-induced secretion of TNF-α, IL-6, IL-10 or IL-8 (Fig 6B and S8D Fig), which was not surprising since Mav lacks the ESX-1 secretion system and does not translocate into the cytosol where these PRRs are located [6,40]. We confirmed that each siRNA was specific by assessing the expression of both targeted and non-targeted genes (S6D and S8C Figs), and functional by challenging knockdown macrophages with pure ligands expected to activate the particular pathway(s) or act through other PRRs (S8E and S8F Fig). As expected, LPS-induced TNF/IL-6 was strongly impaired in cells treated with siTLR4, but largely unaffected by siTLR7, siTLR8 or siUNC93B1 (S8E Fig). Correspondingly, responses to CL264 were reduced when knocking down TLR7 and normal in siTLR8 and siSTING treated macrophages (S8E Fig). Mav-induced nuclear translocation of NF-κB and IRF-1 was substantially reduced by knocking down *TLR7*, *8* or *UNC93B1* throughout the time course of 4 hours to 3 days (Fig 6C). The TLR8 ligand CL75 also induced IRF-1 nuclear translocation, confirming IRF-1 is activated by TLR8 engagement (S8F Fig).

**Fig 7:**
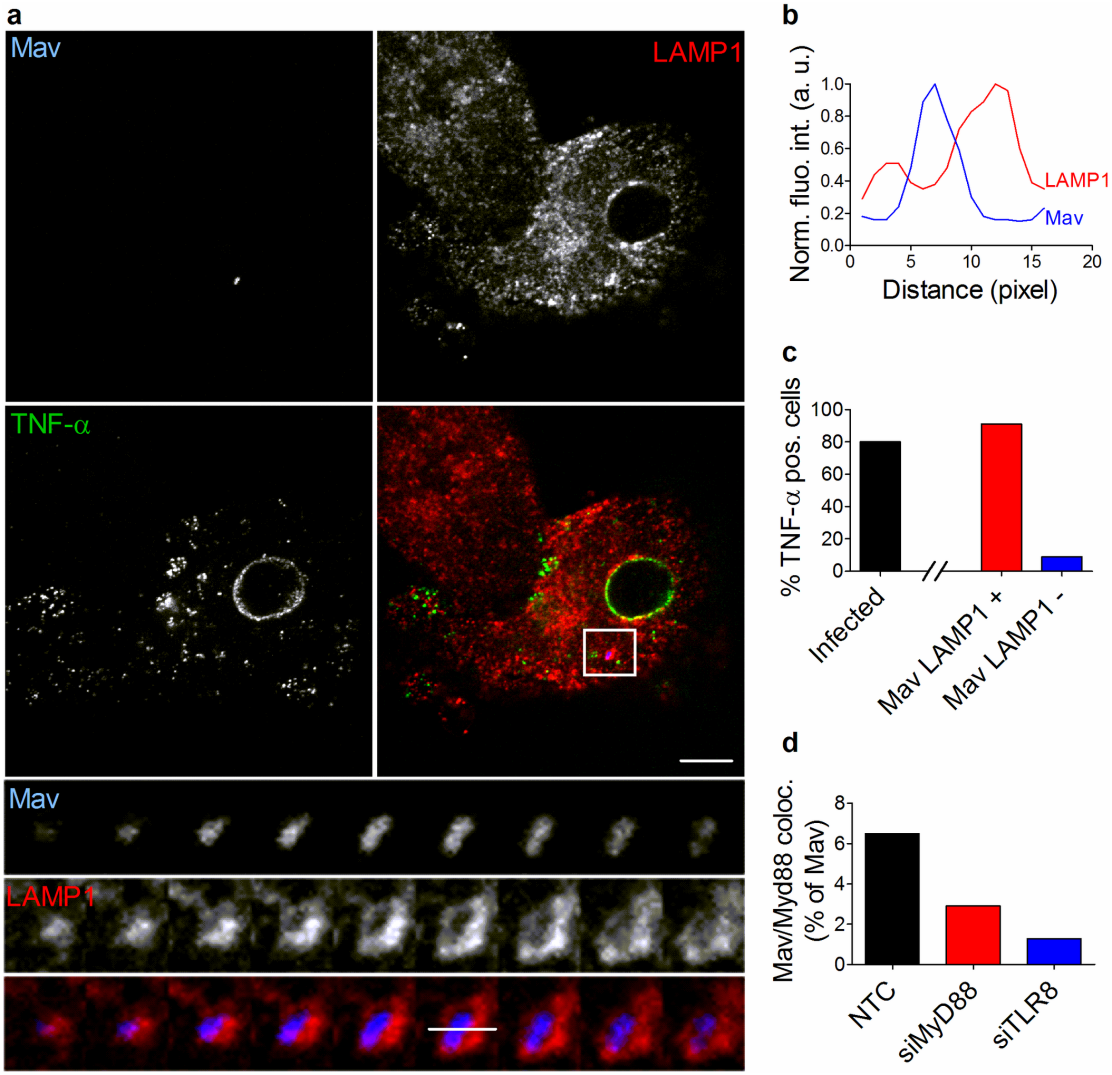
TNF production coincides with phagolysosomal localization of Mav. Human MDMs were infected with live Mav-CFP (blue) for 10 min, chased for 4h in the presence of a protein secretion inhibitor to accumulate cytokines, fixed and stained with antibodies to LAMP1 (red) and TNF (green). (A) Single and merged images of one cell; bottom-to-top projections of 3D-stack from boxed area in lower panels show that Mav is in a LAMP1^+^ compartment. (B) Fluorescence intensity profiles along the indicated lines of the Mav phagosome (Mav: blue trace; LAMP1: red trace). (C) Quantification of the percentage of infected cells secreting TNF (black bar), and of these, the fraction with Mav in LAMP1^+^ compartments (red bar) or LAMP1^-^ compartments (blue bar). Quantification graphs represent the mean value +/- SEM from two different experiments. (D) Human MDMs were treated with siMyD88, siTLR8 or non-targeted control before infection with Mav for 10 min and chase for 4h. Cells were then stained with antibody to MyD88 and analyzed using confocal microscopy for recruitment of MyD88 to Mav phagosomes. Quantification graph represents MyD88-Mav association from two independent experiments. Scale bar represents 10 μm.

We have so far shown evidence that nuclear translocation of transcription factors is only activated from LAMP1^+^ MavPLs, MyD88 is only recruited to MavPLs, and MyD88 is involved in activating cytokine production. We next stained for TNF-α and LAMP1 in Mav-infected macrophages and found that 80% of the infected cells produced TNF-α, and of these, more than 90% harbored Mav in LAMP1^+^ MavPLs whereas less than 10% of the TNF-producing cells had Mav in LAMP1^-^ MavCs (Fig 7A–7C). To detect intracellular TNF-α we used a transport inhibitor cocktail that blocks transport from the endoplasmic reticulum (ER) to Golgi, explaining the accumulation of TNF-α in perinuclear ER (Fig 7A). Taken together these results suggest that Mav engages TLR8 and also TLR7 in phagolysosomes, resulting in recruitment of MyD88, inflammatory signaling and secretion of cytokines.

### TLR7/8/MyD88 are required to control intracellular growth of Mav in human primary macrophages

**Fig 8:**
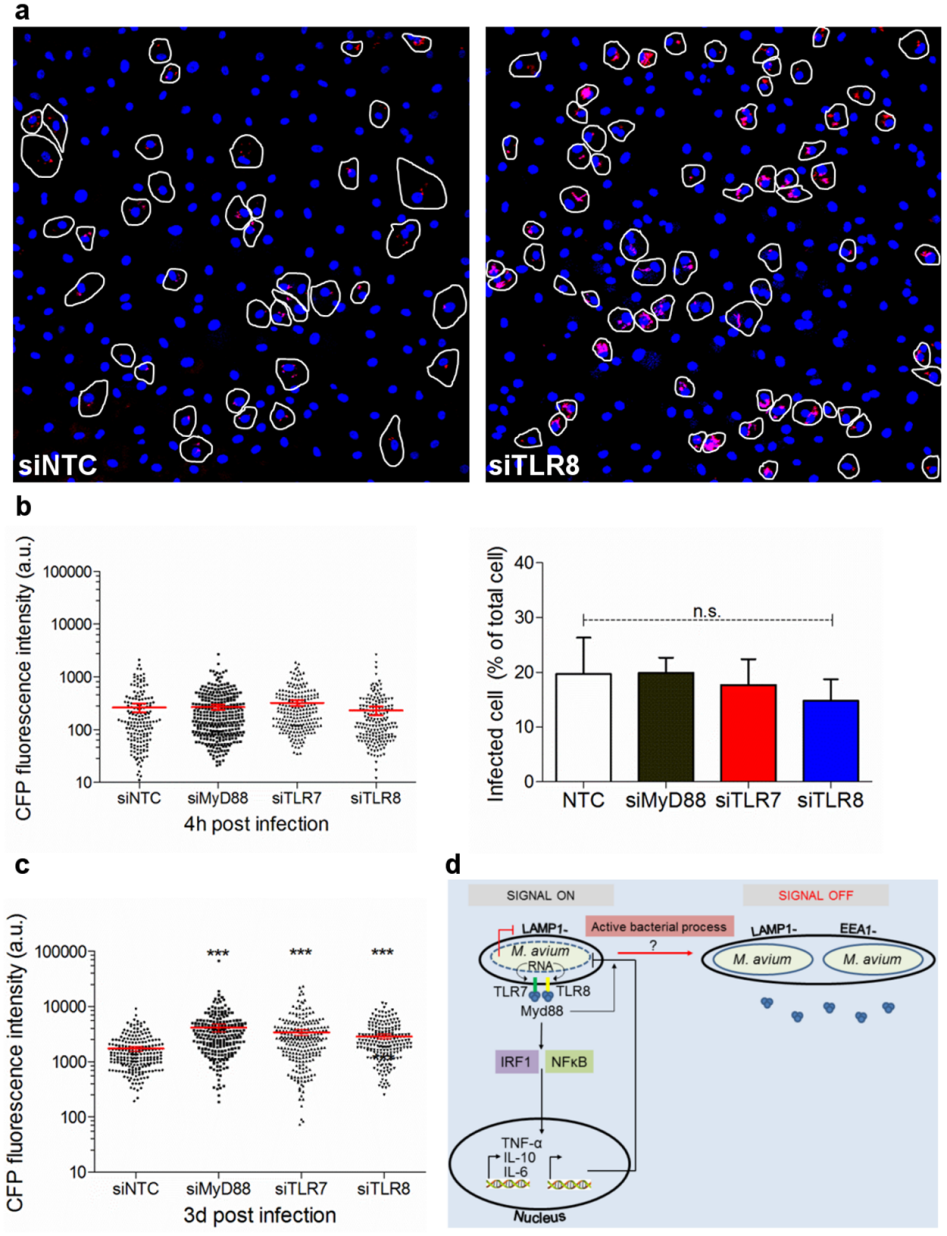
TLR7/8/MyD88 regulate intracellular growth of Mav in human primary macrophages. Human MDMs were treated with siRNA against MyD88, TLR7, TLR8 or a non-targeting control before infection with Mav-CFP (10 min uptake followed by 4h to 3d chase). (A) Representative images where infected cells treated with siNTC (left) or siTLR8 (right) are outlined for analysis (Mav-CFP: red, nuclei: blue (Hoechst)). (B, C) *In situ* quantification of Mav-CFP 4 hours (B) and 3 days (C) post infection using the Corrected Total Cell Fluorescence method on infected cells. Quantification scatter plots show individual cell measures with mean +/- 95% confident intervals from 3 donors ((B, C) left graphs, n>250 cells per time point and per donor). The bar chart in (B) shows the percentage of infected cells 4 hours post infection to compare uptake. (D) Working model. Mav (dashed line, blue) is processed in LAMP1^+^ phagolysosomes. Release of mycobacterial nucleic acids engages TLR7/8, recruitment of MyD88 to the compartment, and inflammatory signaling culminating in cytokine release and growth restriction. A fraction of live Mav actively remodels the phagolysosome and/or is sorted into a new compartment (MavC) where late endosomal/lysosomal markers are excluded, TLR7/8 are not engaged and MyD88 is not recruited, thus avoiding inflammatory signaling and destruction. The program leading to Mav destruction (red line) remains to be elucidated.

TLRs signal via the canonical IKK-complex and we recently showed the importance of IKK-β in controlling intracellular growth of Mav in human primary macrophages [27]. To examine if TLR7/8/MyD88 signaling affects intracellular Mav growth we next quantified Mav-CFP intensities across infected macrophages at the single cell level 4 hours and 3 days post infection (Fig 8). The CFP-level and thus the total number of Mav increased ten-fold over 3 days of infection (Fig 8B and 8C). 4 hours post infection there was no difference in CFP-levels between target or control siRNA treated cells, whereas the CFP-levels were significantly higher in cells treated with siMyD88, siTLR7 or siTLR8 compared to non-targeting control 3 days post infection, suggesting that TLR7/8/MyD88 signaling is central in controlling intracellular growth of Mav in human primary macrophages (Fig 8B and 8C). A working model summarizing our findings in the paper is shown in Fig 8D.

## Discussion

Despite decades of research on mycobacterial phagosome biogenesis the process is incompletely understood, including how mycobacteria interact with and evoke host responses from the various subcellular compartments inside macrophages. Both MavCs and MtbCs are generally found to retain characteristics of early endosomes with elevated pH, unprocessed cathepsins and access to transferrin, suggesting that phagosomal maturation is arrested at an early stage [28–31,36,37,52]. Using confocal microscopy on single-infected human primary macrophages we have revealed here that Mav phagosomes are not arrested at an early endosomal stage but mature normally to LAMP1^+^ phagolysosomes where Mav is degraded and recognized by TLR7/TLR8, eliciting host responses by recruitment of MyD88 to the phagolysosomal compartment. A fraction of Mav escapes phagolysosomal degradation by re-establishing in a new compartment where it replicates and, most importantly, to where MyD88 is not recruited and inflammatory signaling is not evoked. This way Mav eventually evades both degradation and inflammatory host responses that could be detrimental for intracellular survival and establishment of chronic infection.

Our findings are not all contradictory to published data: several studies show LAMP1/LAMP2 or CD63 staining of mycobacterial compartments to varying degrees [28–31,36–38,52,53]. These have often been interpreted to contain dead bacteria, or in the cases where the proton-ATPase is also lacking, not considered to be fully phagolysosomal in nature. However, spearheaded by electron microscopy studies of macrophages infected with Mav, Mtb or *M*. *bovis* BCG, compelling evidence indicates that mycobacteria transit into late endosomes/lysosomes before re-establishing a new compartment [33,38,54–57]. This new compartment would be equivalent to our MavC where we see no LAMP1/LAMP2/CD63, no MyD88 recruitment or signaling, but mycobacterial division. It has been observed that when mycobacteria divide, the vacuole appears to divide with them, sequestering each bacillus in a separate tight vacuole [52,54,58]. Tight membrane apposition may facilitate their control of fusion with other vacuoles within the cell and mycobacterial cell wall lipids are shown to be critical in maintaining the MtbCs [29,38]. Our previous studies showed that MavCs are positive for transferrin and Rab11a suggesting communication with recycling endosomes [34], also in agreement with literature [36,53].

The lack of MyD88 recruitment and inflammatory signaling from the MavC suggest this compartment is key to survival and growth of Mav inside macrophages, and thus an attractive therapeutic target. However, several questions remain to be answered: How are mycobacteria sorted into MavCs and prevent them from fusing with lysosomes? Moreover, given McDonough and others are right in their observations that MavCs and MtbCs bud off from the phagolysosome: are TLRs or sorting TLR adaptor TIRAP [59] excluded from the membrane of forming MavCs, or is the absence of Mav recognition and MyD88 recruitment to the MavC due to lack of either ligand exposure (no RNA or DNA release) or ligand binding to TLRs? For the first question our results suggest Mav needs to be viable to establish in MavCs since PFA-killed Mav were retained in phagolysosomes. This is also well described in literature and stress factors like low pH, oxidative stress and nutrient deficiency in the maturing phagosome may act as important cues to the bacteria enabling them to mount counteractive mechanisms and escape [1,29]. Several virulence factors are needed for intraphagosomal survival [57,60–66], but only a few secreted phosphatases (PtpA, SapM [67–69]) and a phospho-inositide 3 kinase mimic (MAV_2941 [70]) are so far shown to directly impact on the fusogenicity of the compartment. How these virulence factors access the cytosol is unclear, although ESAT-6, an Mtb antigen that is secreted through the ESX-1 secretion system, is shown to have pore-forming activity. ESX-1 is also needed for the release of Mtb DNA and translocation of Mtb into the cytosol during infection [39,71]. Mav lacks the ESX-1 secretion system and does not translocate into the cytosol [28,40,72]. However, Bermudez' group recently identified an Mav oligopeptide transporter, OppA, that delivers MAV_2941 into the macrophage cytosol to interfere with phagosomal maturation [70,73]. We did not find STING or NOD1 to be involved in Mav inflammatory responses, nor did we observe any macrophage cell death indicative of inflammasome activation and pyroptosis, suggesting little to no Mav ligands were translocated into the cytosol to activate the cytosolic surveillance system. Our results thus support a scenario where Mav phagosomes mature to a late endosomal/lysosomal stage where decreasing pH and other intraphagosomal cues induce stress-responses in the mycobacteria facilitating their re-establishment in the MavC.

Our discovery that pathogenic mycobacteria hide and divide in a compartment that is devoid of TLR signaling is significant. For endosomal TLR signaling to happen, the receptors need to be trafficked to the right compartment and proteolytically cleaved to bind ligands. Activated ligand-TLR dimer complexes are recognized by sorting adaptors TIRAP or TRAM that recruit signaling adaptors MyD88 or TRAM to form a supramolecular organizing center (e.g. Myddosomes) conveying signaling [11,23,24]. UNC93B1 mediates the trafficking of TLR3, 7, 8 and 9 to endosomes and the absence of UNC93B1 abrogates signaling [11], which is what we also observed for Mav in siUNC93B1 treated macrophages. The mechanism by how TLR7/8 are recruited to Mav containing phagolysosomes is elusive, if it is induced by Mav recognition on the plasma membrane or during phagocytosis, and if it involves regulated trafficking and fusion of preformed TLR7/8 endosomes with the Mav containing phagosomes. Human TLR7 and TLR8 are processed at neutral pH and may be present in MavCs, but receptor engagement most likely happens in lysosomal compartments since both TLR7 and 8 respond to ssRNA degradation products (ribonucleosides and oligoribonucleotides) and not intact ssRNA, indicating RNA processing should be required [14–18]. Regarding the second question, this could explain why Mav does not signal from the MavC, which is not acidified and thus have limited proteolytic activity, together with the fact that live and intact Mav probably does not release much nucleic acids. We did not observe Myddosome formation on MavCs, and work is in progress to verify if signaling is not emerging from MavCs because TLRs are not activated or if TLRs or sorting/bridging adaptors are excluded from the compartment.

Instead we found that Mav was degraded and engaged TLR7/8/MyD88 in the phagolysosomal compartment, triggering Myddosome formation, cytokine release and Mav growth restriction. Concomitant engagement by both TLR7 and TLR8 is not surprising since one would expect most microbes to contain ligands for both receptors. TLR7 and 8 recognize ssRNA degradation products together with guanosine or uridine nucleosides, respectively, in a species-specific manner [14–18,50]. However, it is unclear why two ssRNA receptors have evolved and, whereas some cells like human pDCs predominantly express TLR7, we found both TLR7 and TLR8 to be present in human primary macrophages (here and [19]). Furthermore, although TLR8 was more reliably involved in activating Mav cytokine responses, knocking down either receptor resulted in substantially reduced cytokine responses by most donors and increased intracellular growth of Mav. Our findings are partly in agreement with a study by Mancuso et al. describing activation by group B streptococci of a TLR7/MyD88/IRF1 pathway driving IFNβ expression in mouse conventional DCs, and where MyD88 was recruited to LAMP1^+^ compartments containing partly digested bacteria [22]. We found that Mav engagement of TLR7/8/MyD88 activated nuclear translocation of NF-κB and IRF-1, and induction of inflammatory cytokines. Our previous studies have indicated a possible involvement of IRF-1 and IRF-5 in Mav-induced IFNβ-responses [27], and *Staphylococcus aureus* RNA induces IFNβ in a TLR8/IRF-5 dependent manner [19]. However, Mav is a weak inducer of type I IFNs in human primary macrophages despite pronounced IRF-1-activation, and discrepancies could thus arise from differences in species, cell types, and the pathogens investigated. IRF-1 is strongly induced by interferons but can also be directly recruited to promoter elements of TNF, IL-6 and IL-12β by LPS stimulation [47,74]. IRF-1 is also involved in TLR2-mediated induction of CCL5 in mouse macrophages [75] and viral induction of type III IFNs in epithelial cells [76]. We show activation of IRF-1 nuclear translocation by the TLR8 ligand CL75 and have previously shown that Mav-induced IRF-1 involves IKKβ, but its role in mycobacterial infections remains to be established.

Heterogeneity is usually reported for mycobacterial phagosomes regarding membrane markers and organelle identity, underlining the need for careful spatiotemporal studies of mycobacterial trafficking in the subcellular space of single cells. These are hampered by the scarcity of good antibodies for use in primary cells and the dangers associated with overexpression of fluorescently tagged proteins in cell lines. Moreover, it is notoriously difficult to probe the viability of single mycobacteria: slow growth rates, thick and impermeable cell wall and apparent persistence of reporter proteins even post mortem is commonly experienced, making it difficult to discriminate recently dead from live bacteria. Based on our findings we expect the phagolysosome to contain a mixture of live and dead Mav at all times. Over time we observed morphological changes indicative of mycobacterial degradation in LAMP1^+^ phagolysosomes and, conversely, elongated mycobacteria in division in LAMP1^-^ MavCs, but never the other way around. We also confirmed that Mav replicates in MavCs and not in MavPLs by counting bacteria in LAMP-stained compartments over time. We thus feel confident that the MavC supports mycobacterial growth. Still it remains to be proved exactly how Mav modulates host membranes to prevent fusion with lysosomes and either excludes TLRs or interferes with recognition and signaling to facilitate survival.

## Materials and methods

### Reagents

The following primary antibodies were used: anti-EEA1 (Santa-Cruz, H300), anti-IRF-1 (Santa-Cruz, C20), anti phospho-p65 (CST, XP(R) D14E12), anti-LAMP1 mouse monoclonal (Santa-Cruz, H4A3) and rabbit polyclonal (Abcam, ab24170), anti-CD63 (Abcam, ab59479) and anti-MyD88 (Abcam, ab2064). Secondary antibodies Alexa Fluor 555 or Alexa Fluor 647–conjugated goat anti-rabbit or anti-mouse IgG and the nuclear dye Hoechst 33342 were from Life Technologies. Ultrapure LPS (*E*. *coli* 0111:B4), TLR7 ligand CL264 and TLR8 ligand CL75 were purchased from Invivogen.

### Isolation and differentiation of human primary macrophages

Human peripheral blood mononuclear cells (PBMCs) were isolated from buffy coats obtained from the Blood Bank, St Olav’s Hospital, Trondheim, Norway, by density centrifugation using Lymphoprep (Axis-shield). Monocyte-derived macrophages (MDMs) were generated by plastic adherence for 1h in complete RPMI 1640 (680 μM L-Glutamine and 10 mM Hepes, GIBCO) supplemented with 5% pooled human serum (The Blood Bank, St Olavs hospital) at 37°C and 5% CO_2_. After three washing steps with Hank’s Balanced Salt solution (GIBCO), monocytes were cultivated for 6 days with a change of medium at day 3 in RPMI 1640/10% human serum and 10 ng/ml recombinant M-CSF (R&D Systems). At day 6 the medium was replaced with RPMI 1640/10% human serum and used for experiments on day 7.

### siRNA transfection of MDMs

Transfection with siRNA was performed using siLentFect Lipid Reagent (Bio-Rad) for RNAi according to the manufacturer’s protocol. Gene knockdown was evaluated by reverse transcription quantitative PCR (RT-qPCR). MyD88, UNC93B1, TLR4, TLR7, TLR8, NOD1 and STING pooled ON-TARGETplus human siRNAs (Dharmacon/Thermo Scientific) were used to target *MyD88*, *UNC93B1*, *TLR4*, *TLR7*, *TLR8*, *NOD1* and *STING*. MDMs were treated with 20 nM siRNA two times (day -4 and day -2) before changing to fresh medium (RPMI 1640/10% human serum), rest for 1–2 hours and challenge with TLR ligands or Mav.

### RNA extraction and RT-qPCR analysis of mRNA levels

MDMs were washed with cold PBS and lysed in buffer RLT (Qiagen) with 1% β-mercaptoethanol. Total RNA was extracted using RNeasy Mini kit and QIAcube according to the manufacturer’s protocol (Qiagen), including DNase I digestion (RNase-free DNase set). The samples included in the study presented an OD_260/280_ ratio ~ 2 assessed using a ND-1000 spectrophotometer (NanoDrop). cDNA was synthetized from normalized amounts of RNA using the High-Capacity RNA-to-cDNA kit according to manufacturer’s recommendations (Applied Biosystems). qPCR reactions were performed in 20 μl total volume with 10 ng cDNA input, PerfeCta qPCR FastMix, UNG, ROX (Quanta Biosciences) and TaqMan Gene Expression Assays (Applied Biosystems): GAPDH (Hs99999905_m1), TLR7 (Hs019333259_s1), TLR8 (Hs00152972_m1), MyD88 (Hs00182082_m1), UNC93B1 (Hs00276771_m1), STING (Hs00736958_m1), NOD1 (Hs00196075_m1). The targeted genes were amplified with a StepOnePlus Real-Time PCR System and relative quantities of gene expression were calculated using the comparative C_T_ method with GAPDH gene expression as endogenous control.

### Mav culture, macrophage infection and challenge with TLRs ligands

Mav clone 104 expressing CFP was cultured in liquid Middlebrook 7H9 medium (Difco/Becton Dickinson) supplemented with 0.5% glycerol, 0.05% Tween 80 and 10% albumin dextrose catalase. Cultures were maintained at log phase growth (optical density between 0.3 and 0.6 measured at 600 nm, OD600) in a 180 rpm shaking incubator at 37°C for a maximum of 5 days. At the day of infection, bacteria were washed with PBS, sonicated and passed through a Gauge 15 needle to ensure single-cell suspension before challenging day 7 MDMs for 10 minutes at a multiplicity of infection of 10. MDMs were subsequently washed and maintained in culture for the appropriate time. Time course of infection was carried out in a backward manner (i.e. first infection corresponding to day 3 post infection, second infection corresponding to day 2 post infection etc) in order to process all samples for immunostaining at the same time. In some experiments MDMs were challenged with TLR ligands for 4 hours at the following concentration: ultrapure LPS (TLR4, 25 ng/ml), CL75 (TLR8, 1 μg/ml), CL264 (TLR7, 2.5 μg/ml).

### Immuno-staining

Human MDMs cultivated on glass-bottomed 96 well plates (IBL) were fixed and permeabilized using a standard protocol as previously described [77]. Briefly, cells were fixed in 4% PFA for 10 min and then incubated in NH_4_Cl for 10 min to quench PFA-induced auto-fluorescence prior to permeabilization with PBS/0.05% Saponin. Cells were next incubated for at least 90 min in PBS/0.05% Saponin/20% human serum to reduce non-specific binding before staining with primary antibodies (1 μg/ml) in PBS/0.05% Saponin/1% human serum over night at 4°C. Cells were washed with PBS/0.05% Saponin/1% human serum and incubated with secondary antibodies for 45 min at room temperature, washed again and stored at 4°C in PBS containing Hoechst for nuclear staining.

### Confocal imaging

For resolution limited imaging, MDMs cultivated on glass-bottomed 96 well plates were imaged either with a Zeiss LSM510 confocal microscope with 63x NA = 1.4 objective (Carl Zeiss Micro-imaging Inc.) or with a Leica SP8 confocal microscope (Leica Microsystem) with 63x NA = 1.4 objective. Emissions were collected using PMT detectors (Zeiss LSM510) and HyD detectors (Leica SP8). In both cases the following acquisition parameters were used: numerical zoom set to reach a pixel size of approximately 90 nm, frame averaging 4, and 3D acquisition to collect the entire cell with a z step of 0.3 μm. Each fluorophore was recorded using sequential acquisition to minimize cross excitation and channel bleed through. On Zeiss LSM510 Hoechst was excited with a 405 nm diode laser and emission was collected through a 420–480 IR band-pass filter. CFP was excited with a 458 nm Argon laser and emissions were collected through a 470–500 band-pass filter. Alexa-555/633 fluorophores were excited with 565/633 nm HeNe lasers and emissions were collected through a 575–615 band-pass filter and a 650 long-pass filter, respectively. On the Leica SP8 microscope, Hoechst and CFP were excited using a 405 nm diode laser and emissions collected by adjusting detector windows as follows: Hoechst 420–470 nm and CFP 480–540 nm. Alexa-555/633 fluorophores were excited with 553/638 nm diode lasers and collection windows were set between 560–630 nm and 650–750 nm, respectively. The same parameters were used for non-resolution limited imaging with the following changes: 20x NA = 0.5 or 40x NA = 1.3 objectives, no numerical zoom was applied, frame averaging 2, 3D acquisition was recorded with a z step of 0.7 μm.

For phagosomal acidification, MDMs previously infected with Mav-CFP were loaded with Cresyl Violet (Sigma-Aldrich) 1 μM diluted in complete media for 5 min at 37°C and then washed with fresh media. Cells were imaged with Zeiss LSM510 confocal microscope with 63x NA = 1.4 objective (Carl Zeiss Micro-imaging Inc.) equipped with a 37°C encagement. Cresyl Violet was excited with 547 laser diode and emissions were collected through a 575 long-pass filter.

### Image analysis

Images were analyzed with Image J (NIH) and Leica Analyzing Suite (Leica Microsystems). High-resolution images were screened in 3D, Mav localization in early endosomes (EEA1) and lysosomes (LAMP1, CD63) was assessed by quantifying fluorescence levels along a line manually placed to intersect bacterial phagosomes using the “Plot Profile” tool in Image J. For quantification of nuclear translocation, single fluorescence images and merged images were synchronized and nuclear localization of the transcription factors assessed based on Hoechst staining. Cells on edges of the field of view (i.e. not completely acquired in the FOV) were taken into account only if bacteria and nuclei were detected. Cells were classified as follows: the total number of cells, number of infected cells and number of activated cells (i.e. with transcription factor in the nucleus). The percentage of each category for one specific time point and one specific donor was calculated for each field of view and compared to the control.

For the MyD88 aggregation assay, images were treated as follows: MyD88 nuclear staining, if any, was removed; aggregates were segmented with the “Threshold” algorithm from Image J using Max “Entropy option” and with the fluorescence intensity defined by the control condition. Aggregates were counted using the “Find Maxima” application.

### In situ CFU measurement

3D stacks were projected using the “average” setting. Cells containing Mav as well as background regions were manually encircled and CFP levels (integrated density) were measured. Corrected Total Cell Fluorescence (CTCF) was calculated using the following formula: CTCF = Integrated Density–(Area of selected cell x Mean fluorescence of background). A minimum of 250 infected cells per condition and per donor were counted.

### Cytokine measurements

Supernatants from human MDMs challenged with Mav or TLR ligands were collected and cytokine secretion profiles for GM-CSF, GRO-α, IFN-α/β/γ, IL-1α/β, IL-1 Receptor Antagonist, IL-2 IL-4, IL-5, IL-6, IL-8, IL-10, IL-12p70, IL-17A, IL-18, IL-23, IL-31, IP-10, MCP-1, RANTES, SDF1-α and TNF-α/β were analyzed according to the manufacturer's instruction using the ProcartaPlex Human Cytokine and Chemokine panel (Affimetrix, eBioscience).

### Statistical analysis

For each donor, the Fisher Exact Test was used to compare measurements at different time points with the control, or to compare live with PFA-killed Mav responses at a given time point. For nuclear translocation experiments, two-tailed *t*-test was used on donor average values. For intracellular localization exact and single donor analysis *P* values were calculated using the Fisher Exact Test. Areas under the curve were calculated for each cytokine/donor couple using the Prism software (Graph Pad) and *P* values were calculated using Wilcoxon matched-pairs rank test. Significant *P* values were set as follows: * <0.05, ** <0.01 and *** <0.005.

## Supporting information

S1 Fig Mav is targeted to CD63^+^/LAMP1^+^/LAMP2^+^ compartments. Human MDMs were challenged with Mav-CFP (blue) for 10 min, chased for 4h to 3d, stained with antibodies to LAMP1 (green) and CD63 (red, (A)) or LAMP2 (red, (D)) and analyzed using confocal microscopy. Single labeling and merged images of a macrophage harboring Mav inside a LAMP1^+^CD63^+^ (A) or LAMP1^+^LAMP2^+^ (D) late endosome/lysosome are shown. Bottom-to-top projections of 3D-stacks from boxed area in lower panels represent Mav, LAMP1, CD63 or LAMP2, and merged images. Quantification of Mav associated with LAMP1^+^CD63^+^ (B) or LAMP1^+^LAMP2^+^ (E) compartments was performed by fluorescence intensity profiling along indicated lines of Mav phagosomes (Mav: blue trace; LAMP1: green trace; CD63 or LAMP2: red trace. (C). Quantifications of the percentage of Mav in LAMP1^+^CD63^+^ compartments. For each time point, at least 40 cells were recorded per donor for two donors. Quantification graphs represent mean value +/- SEM of Mav localization in LAMP1^+^CD63^+^ compartments. *P* values were calculated using Fisher Exact test for each single donor (* *P* <0.05, ** *P* <0.01 and *** *P* <0.005). a.u.: arbitrary unit. Scale bar represents 10 μm.
(TIF)

S2 Fig Mav is targeted to acidic compartments. Human MDMs were challenged with Mav-CFP (blue) for 10 min, chased for 4h to 3d, and loaded with Cresyl violet for 5 min before confocal microscopy. (A) Single labeling and merged images of Mav-CFP and Cresyl violet are shown. Scale bar represents 10 μm. Magnification of dashed box areas are shown as inserts. Middle panel: The arrow indicates the presence of a weakly fluorescent Mav-CFP (associated magnification in insert). (B) Quantification graph represents mean value +/- SEM of Mav localization in acidic compartments for 2 donors.
(TIF)

S3 Fig PFA treatment kills Mav without affecting infection rates. Mav grown to log-phase (optical density at 600 nm of 0.45) were spun down and treated 10 min with PFA 4%. Live and PFA-killed Mav (black and red, respectively) were cultured and optical density was measured at the indicated times ((A), average of 4 different experiments). (B) Human MDMs were exposed to live or PFA-killed Mav-CFP (10 min uptake followed by chase for 4h to 3d), fixed with PFA 4% and analyzed using confocal microscopy. Infection rates were calculated and averages +/- SEM from 3 different experiments are shown. *P* values were calculated using two-tailed t-test (* <0.05).
(TIF)

S4 Fig Mav-induced nuclear translocation of NF-κB and IRF-1 and secretion of inflammatory cytokines. (A-C) Human MDMs were infected with live Mav-CFP for 10 min followed by chase for up to 3 days. Nuclear translocation of NF-κB and IRF-1 was analyzed by confocal microscopy at the indicated time points using anti-p-p65 and anti-IRF-1 antibodies together with a nuclear stain (Hoechst). Quantification bar-charts represent the mean value +/- SEM of nuclear p-p65 (B) or IRF-1 (C) for each time point (n>600 cells per time point and per donor) for at least 4 donors. *P* values between Ctrl and each time point were calculated using two-tailed t-test (* <0.05, ** <0.01 and *** <0.005). (D) Human MDMs were exposed to live (black line) or PFA-killed (red line) Mav-CFP (10 min uptake followed by chase for up to 3 days) and secreted cytokines assessed by multiplex ELISA. Shown are averages with SEM from 7 donors.
(TIF)

S5 Fig TLR7/8/MyD88 trigger inflammatory signaling induced by PFA-killed Mav. Human MDMs were treated with siNTC (non-targeting control), siMyD88, siTLR7, siTLR8 or siUNC93B1 before infection with PFA-killed Mav-CFP for 10 min, chased for 4h to 3d. Graphs represent TNF-α, IL-6, IL-10 and IL-8 secretion from triplicate wells with cells from one donor.
(TIF)

S6 Fig siRNA treatment efficiency and specificity. Human MDMs were left untreated or treated with siRNA against *MyD88*, *TLR7*, *TLR8* or *UNC93B1* (black, red, blue and cyan, respectively) or non-targeting control (white) and mRNA expression assessed by RT-qPCR. (A, B) Ct values are shown to indicate expression levels for *MyD88*, *TLR7*, *TLR8* and *UNC93B1* in untreated cells (A) and unaffected levels of *GAPDH* in cells treated with the various target siRNAs (B). (C) Knockdown efficiency of target siRNAs. Expression levels of target genes in cells treated with target siRNA relative to control siRNA and after normalization to *GAPDH*. Quantification graphs represent the mean +/- SEM of 4 different experiments. (D) Specificity of siRNA treatment. Relative mRNA expression of “non-targeted genes” in each “targeted gene” condition. Quantification graphs represent one experiment from 3 independent experiments.
(TIF)

S7 Fig Mav induces inflammatory signaling by TLR7/TLR8/MyD88. Human MDMs were treated with siRNA against MyD88, TLR7, TLR8 or UNC93B1 before infection with Mav-CFP for 10 min. Supernatants were harvested at different time points post infection and secreted cytokines assessed by multiplex ELISA. Total cytokine secretion from 4 h to 3 d post infection was calculated as area under the curve (AUC) for each cytokine and the Table represents percent increase (red) or decrease (blue) in AUC for target siRNA treated samples compared to non-targeted control for each of 9 or 10 donors as indicated.
(TIF)

S8 Fig Target siRNAs do not affect Mav uptake but specifically reduce cytokine secretion induced by respective TLR ligands. (A, B) Human MDMs were treated with siRNA against *MyD88*, *TLR7*, *TLR8* or *UNC93B1* and infected with Mav-CFP (10 min exposure followed by chase for 4h or 3d), fixed with PFA 4% and analyzed using confocal microscopy. Percentages of infected cells (mean +/- SEM) at 4 hours compare Mav uptake (A) and Mav cell-to-cell spread at 3 days post infection (B). (C, D) Human MDMs were treated with siRNA against *STING* or *NOD1* and infected with Mav-CFP (10 min exposure followed by chase for 4h or 3d). (C) Knockdown efficiency for siSTING and siNOD1. Genes of interest are shown as mRNA fold of induction relative to control and normalized to GAPDH. (D) Cell supernatants were harvested at the indicated time points post infection and cytokine responses from siSTING treated MDMs assessed by multiplex ELISA. Graphs represent average concentrations +/- SEM of TNF-α, IL-6, IL-10 and IL-8 from 4 donors treated with siSTING (red) or non-targeted siRNA (black). (E) siRNA treated cells were challenged with 25 μg/ml LPS or 2.5 μM of the TLR7 ligand CL264 for 4 hours and relative TNF-α and IL-6 expression assessed by RT-qPCR. (F) Human MDMs were treated with 1 μM of the TLR8 ligand CL75 for 4 hours, stained with anti-IRF1 antibodies together with a nuclear stain (Hoechst) and analyzed for nuclear translocation of IRF-1 using confocal microscopy. Quantification graphs represent the mean value (n>700 cells per time point) of one representative donor from three independent experiments. *P* values were calculated using Fisher Exact test (* <0.05, ** <0.01 and *** <0.005.)
(TIF)
