# Correction: Persistent mycobacteria evade an antibacterial program mediated by phagolysosomal TLR7/8/MyD88 in human primary macrophages

**DOI:** 10.1371/journal.ppat.1006712

**Published:** 2017-11-07

**Authors:** Alexandre Gidon, Signe Elisabeth Åsberg, Claire Louet, Liv Ryan, Markus Haug, Trude Helen Flo

An affiliation for the 6th author is not indicated. Trude Helen Flo is affiliated with: Centre for Molecular Medicine Norway, Nordic EMBL Partnership, University of Oslo and Oslo University Hospital, Oslo, Norway.
