# Supplementary figures and images for: Persistent mycobacteria evade an antibacterial program mediated by phagolysosomal TLR7/8/MyD88 in human primary macrophages
Source: PLoS Pathog. 2017 Aug 14;13(8):e1006551. doi: 10.1371/journal.ppat.1006551 (PMC5570494; doi:10.1371/journal.ppat.1006551)

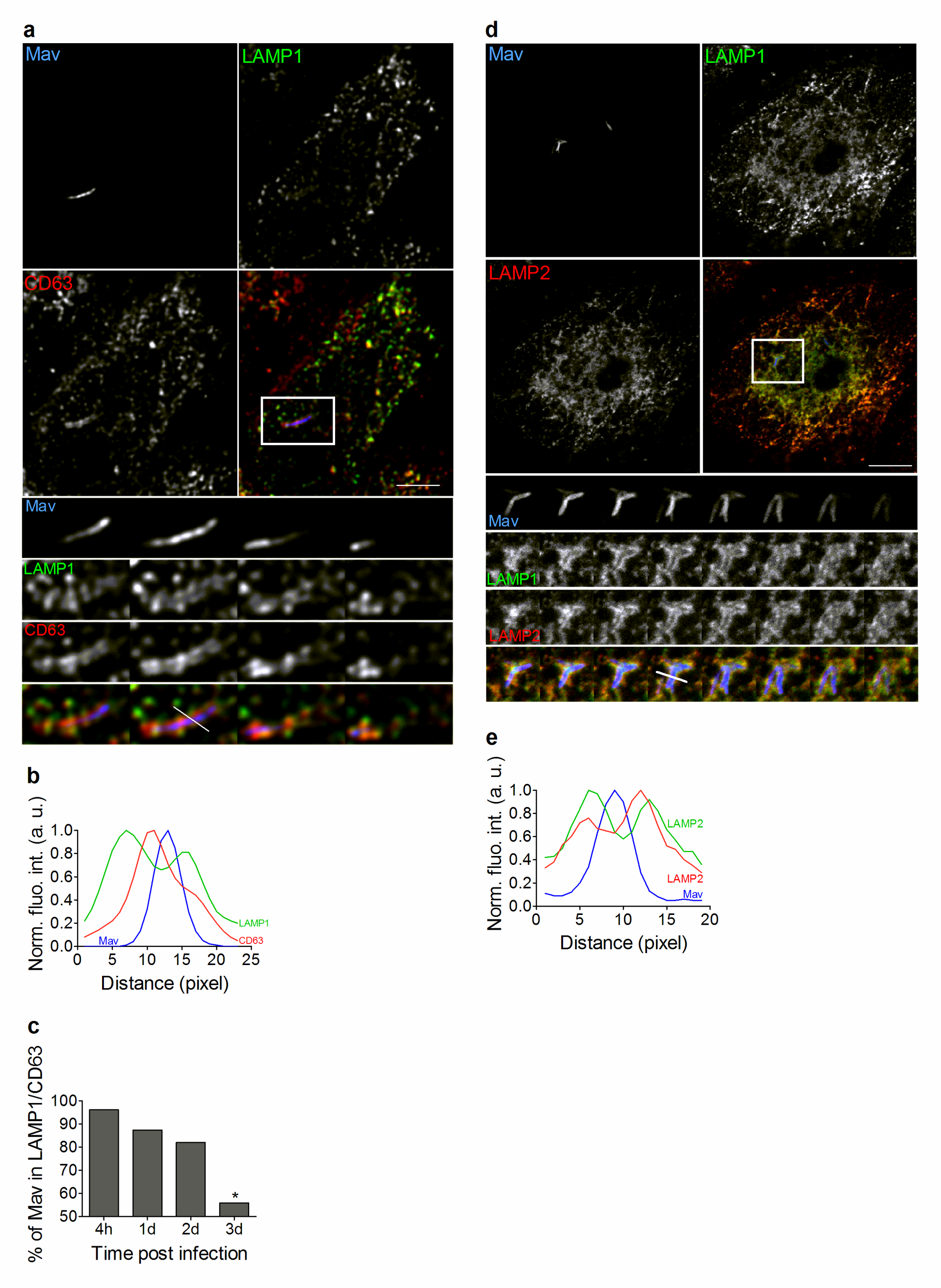

Supplement: S1 Fig — Human MDMs were challenged with Mav-CFP (blue) for 10 min, chased for 4h to 3d, stained with antibodies to LAMP1 (green) and CD63 (red, (A)) or LAMP2 (red, (D)) and analyzed using confocal microscopy. Single labeling and merged images of a macrophage harboring Mav inside a LAMP1+CD63+ (A) or LAMP1+LAMP2+ (D) late endosome/lysosome are shown. Bottom-to-top projections of 3D-stacks from boxed area in lower panels represent Mav, LAMP1, CD63 or LAMP2, and merged images. Quantification of Mav associated with LAMP1+CD63+ (B) or LAMP1+LAMP2+ (E) compartments was performed by fluorescence intensity profiling along indicated lines of Mav phagosomes (Mav: blue trace; LAMP1: green trace; CD63 or LAMP2: red trace. (C). Quantifications of the percentage of Mav in LAMP1+CD63+ compartments. For each time point, at least 40 cells were recorded per donor for two donors. Quantification graphs represent mean value +/- SEM of Mav localization in LAMP1+CD63+ compartments. P values were calculated using Fisher Exact test for each single donor (* P <0.05, ** P <0.01 and *** P <0.005). a.u.: arbitrary unit. Scale bar represents 10 μm. (TIF) [file ppat.1006551.s001.tif]

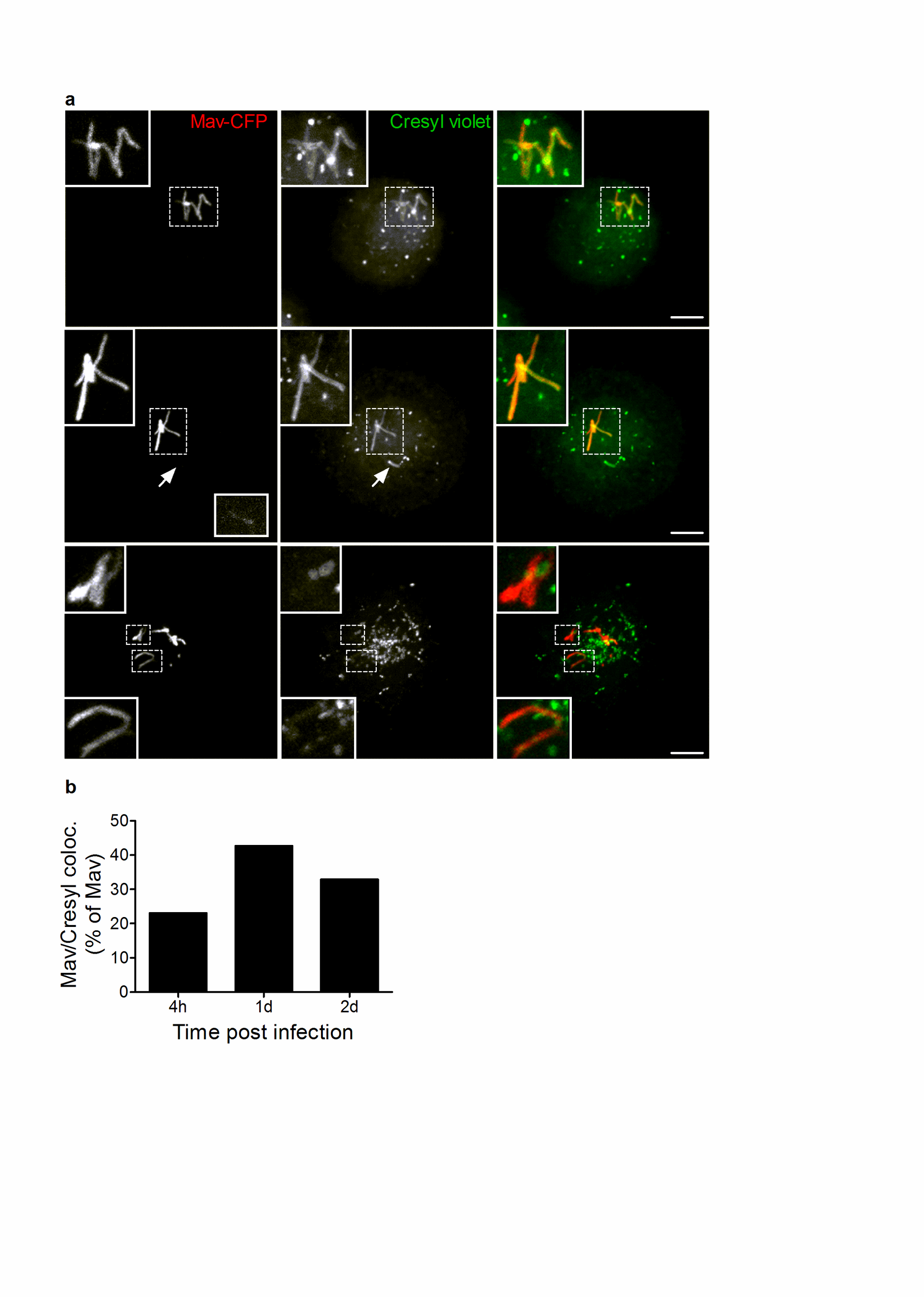

Supplement: S2 Fig — Human MDMs were challenged with Mav-CFP (blue) for 10 min, chased for 4h to 3d, and loaded with Cresyl violet for 5 min before confocal microscopy. (A) Single labeling and merged images of Mav-CFP and Cresyl violet are shown. Scale bar represents 10 μm. Magnification of dashed box areas are shown as inserts. Middle panel: The arrow indicates the presence of a weakly fluorescent Mav-CFP (associated magnification in insert). (B) Quantification graph represents mean value +/- SEM of Mav localization in acidic compartments for 2 donors. (TIF) [file ppat.1006551.s002.tif]

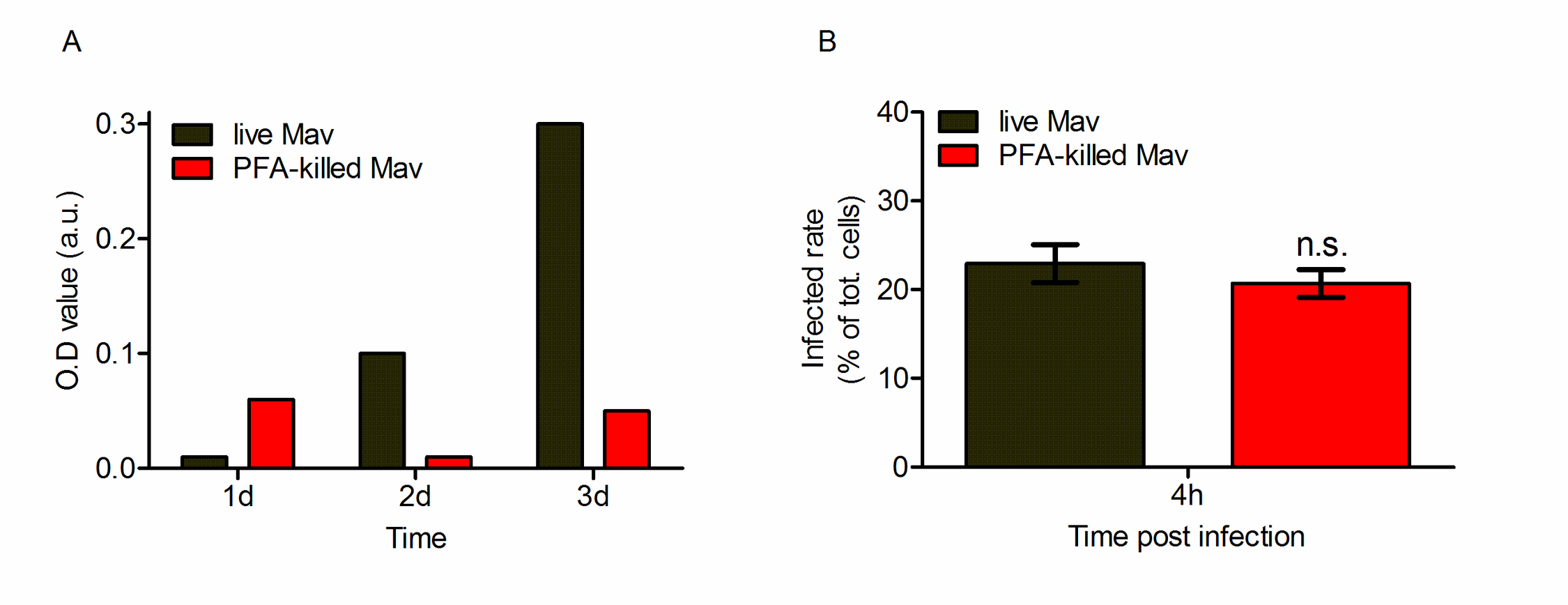

Supplement: S3 Fig — Mav grown to log-phase (optical density at 600 nm of 0.45) were spun down and treated 10 min with PFA 4%. Live and PFA-killed Mav (black and red, respectively) were cultured and optical density was measured at the indicated times ((A), average of 4 different experiments). (B) Human MDMs were exposed to live or PFA-killed Mav-CFP (10 min uptake followed by chase for 4h to 3d), fixed with PFA 4% and analyzed using confocal microscopy. Infection rates were calculated and averages +/- SEM from 3 different experiments are shown. P values were calculated using two-tailed t-test (* <0.05). (TIF) [file ppat.1006551.s003.tif]

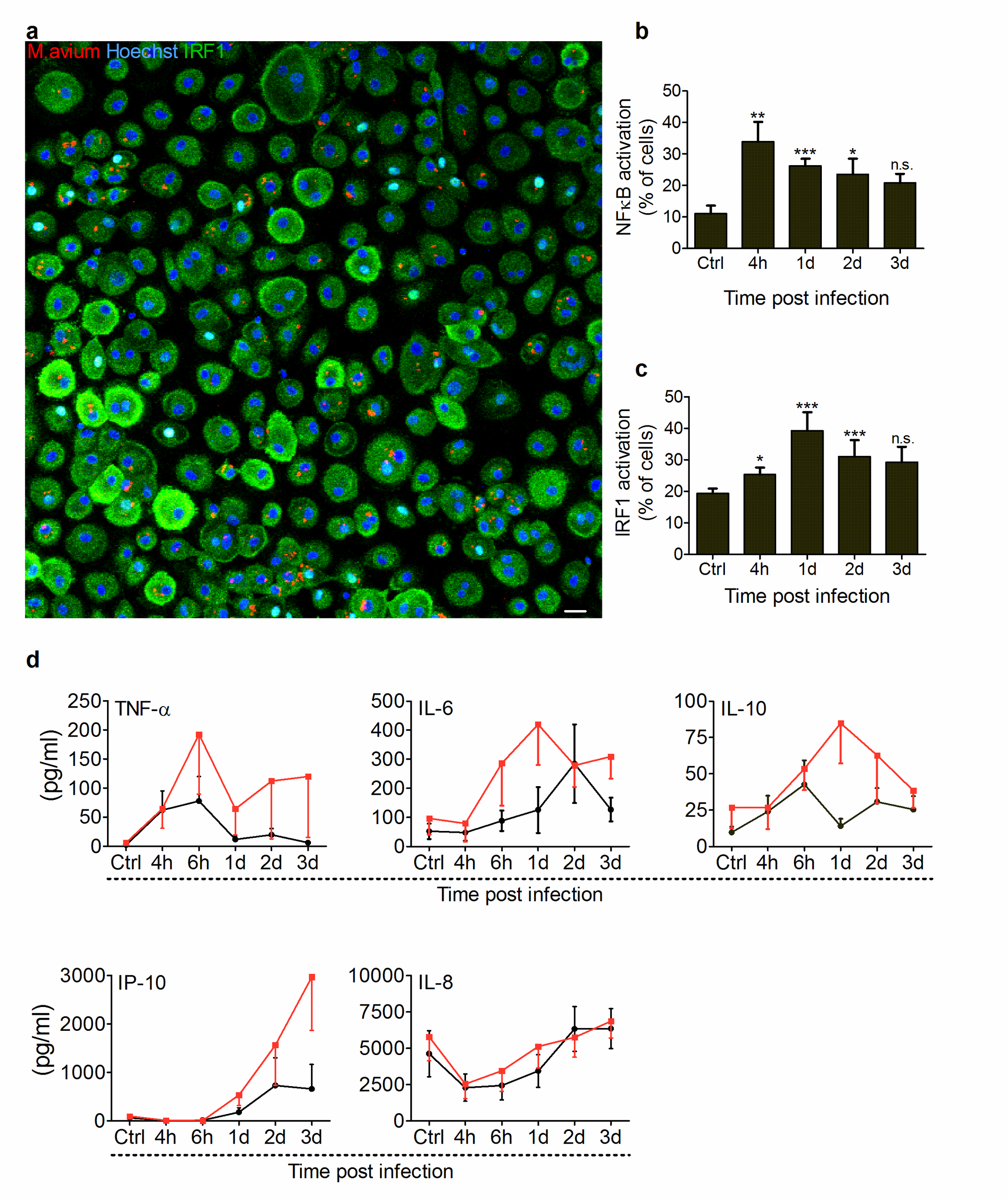

Supplement: S4 Fig — (A-C) Human MDMs were infected with live Mav-CFP for 10 min followed by chase for up to 3 days. Nuclear translocation of NF-κB and IRF-1 was analyzed by confocal microscopy at the indicated time points using anti-p-p65 and anti-IRF-1 antibodies together with a nuclear stain (Hoechst). Quantification bar-charts represent the mean value +/- SEM of nuclear p-p65 (B) or IRF-1 (C) for each time point (n>600 cells per time point and per donor) for at least 4 donors. P values between Ctrl and each time point were calculated using two-tailed t-test (* <0.05, ** <0.01 and *** <0.005). (D) Human MDMs were exposed to live (black line) or PFA-killed (red line) Mav-CFP (10 min uptake followed by chase for up to 3 days) and secreted cytokines assessed by multiplex ELISA. Shown are averages with SEM from 7 donors. (TIF) [file ppat.1006551.s004.tif]

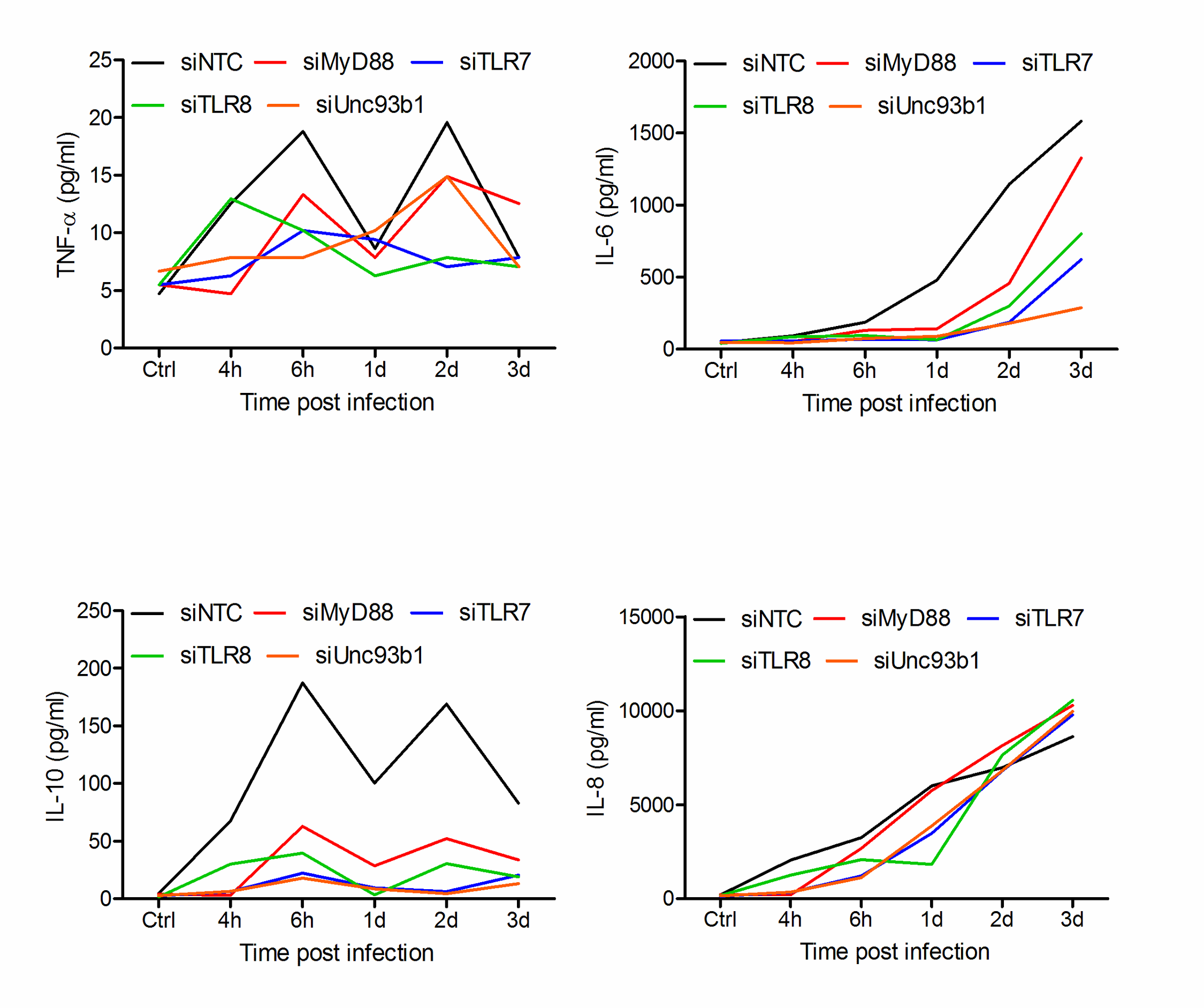

Supplement: S5 Fig — Human MDMs were treated with siNTC (non-targeting control), siMyD88, siTLR7, siTLR8 or siUNC93B1 before infection with PFA-killed Mav-CFP for 10 min, chased for 4h to 3d. Graphs represent TNF-α, IL-6, IL-10 and IL-8 secretion from triplicate wells with cells from one donor. (TIF) [file ppat.1006551.s005.tif]

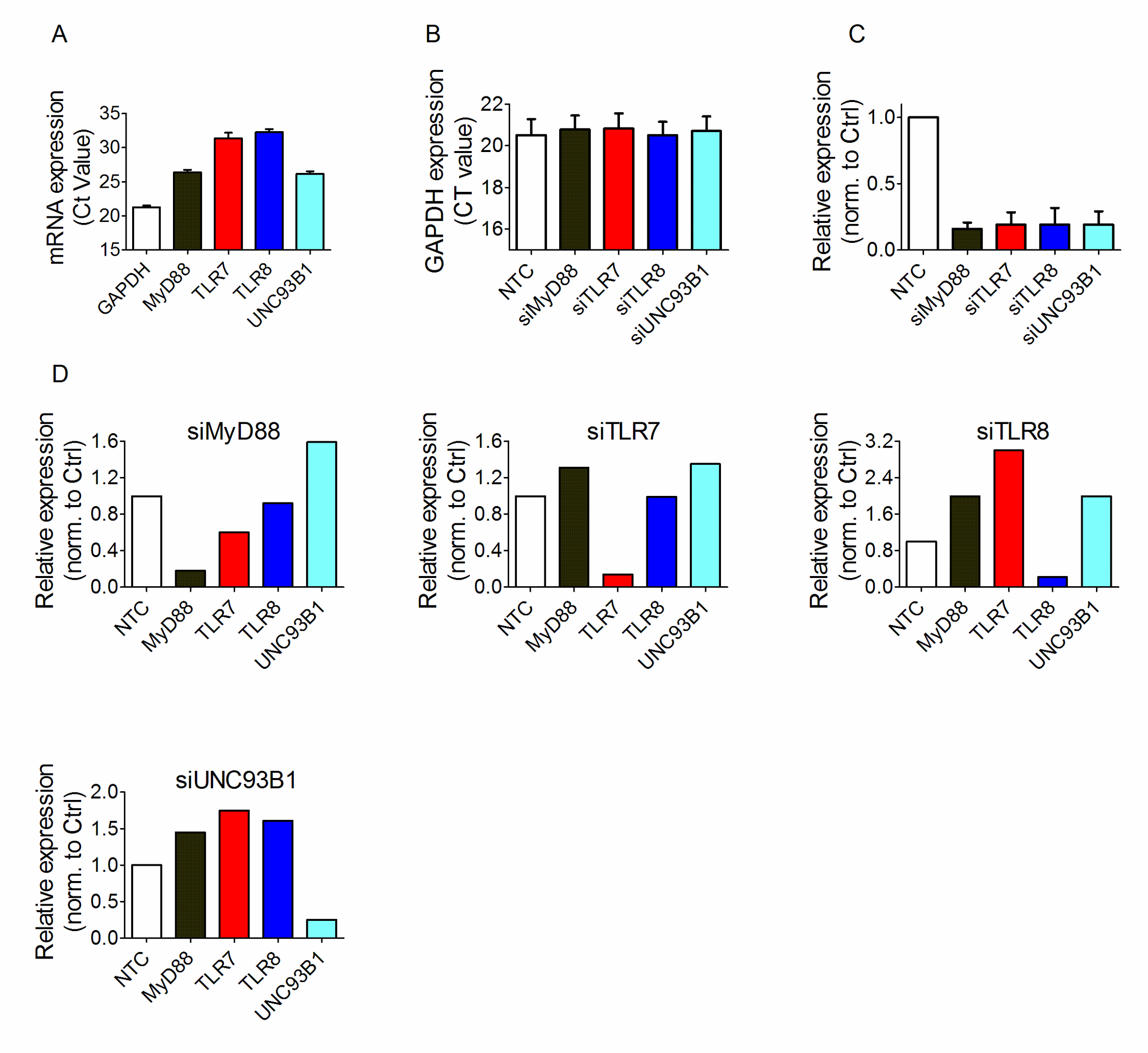

Supplement: S6 Fig — Human MDMs were left untreated or treated with siRNA against MyD88, TLR7, TLR8 or UNC93B1 (black, red, blue and cyan, respectively) or non-targeting control (white) and mRNA expression assessed by RT-qPCR. (A, B) Ct values are shown to indicate expression levels for MyD88, TLR7, TLR8 and UNC93B1 in untreated cells (A) and unaffected levels of GAPDH in cells treated with the various target siRNAs (B). (C) Knockdown efficiency of target siRNAs. Expression levels of target genes in cells treated with target siRNA relative to control siRNA and after normalization to GAPDH. Quantification graphs represent the mean +/- SEM of 4 different experiments. (D) Specificity of siRNA treatment. Relative mRNA expression of “non-targeted genes” in each “targeted gene” condition. Quantification graphs represent one experiment from 3 independent experiments. (TIF) [file ppat.1006551.s006.tif]

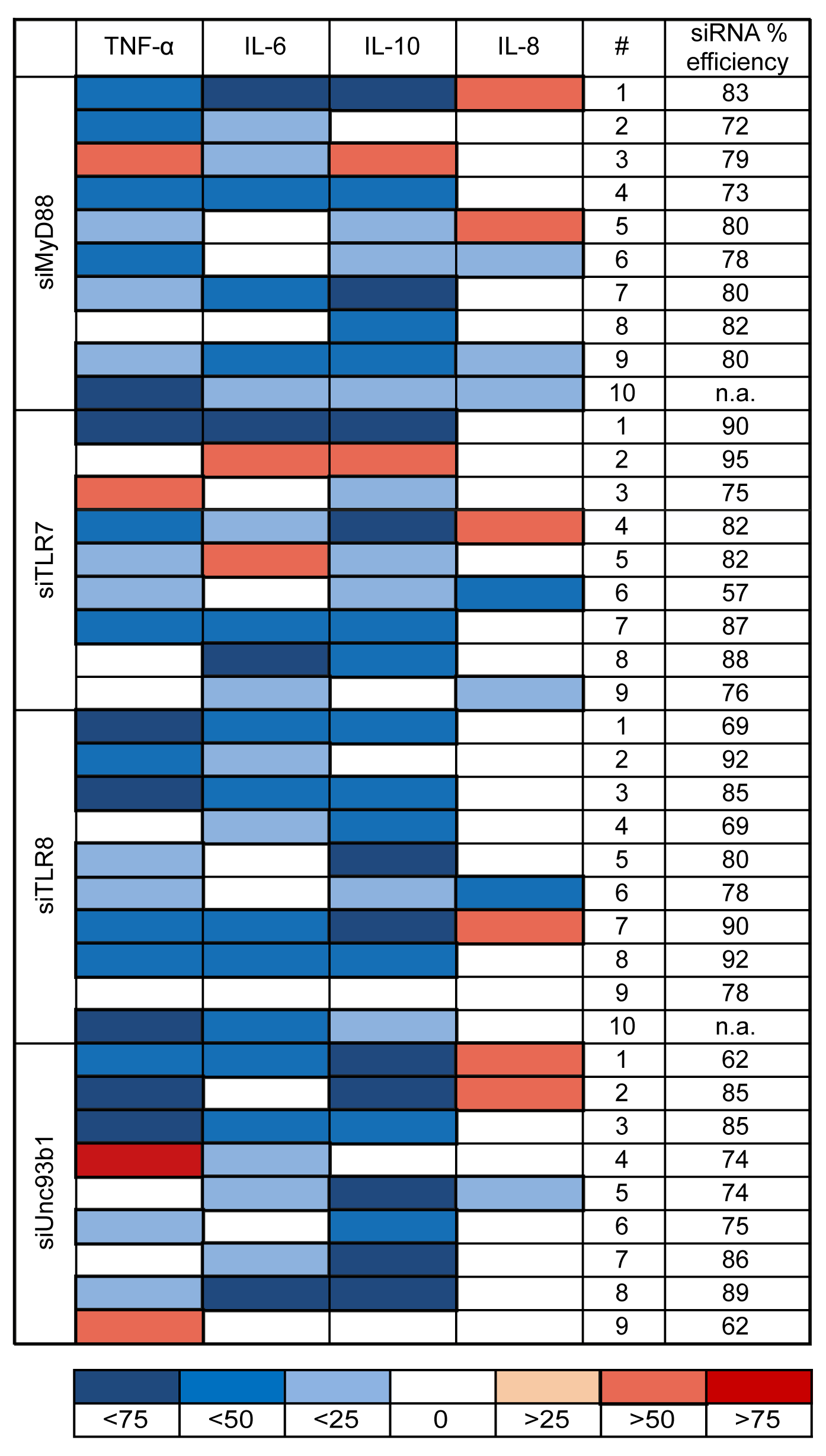

Supplement: S7 Fig — Human MDMs were treated with siRNA against MyD88, TLR7, TLR8 or UNC93B1 before infection with Mav-CFP for 10 min. Supernatants were harvested at different time points post infection and secreted cytokines assessed by multiplex ELISA. Total cytokine secretion from 4 h to 3 d post infection was calculated as area under the curve (AUC) for each cytokine and the Table represents percent increase (red) or decrease (blue) in AUC for target siRNA treated samples compared to non-targeted control for each of 9 or 10 donors as indicated. (TIF) [file ppat.1006551.s007.tif]

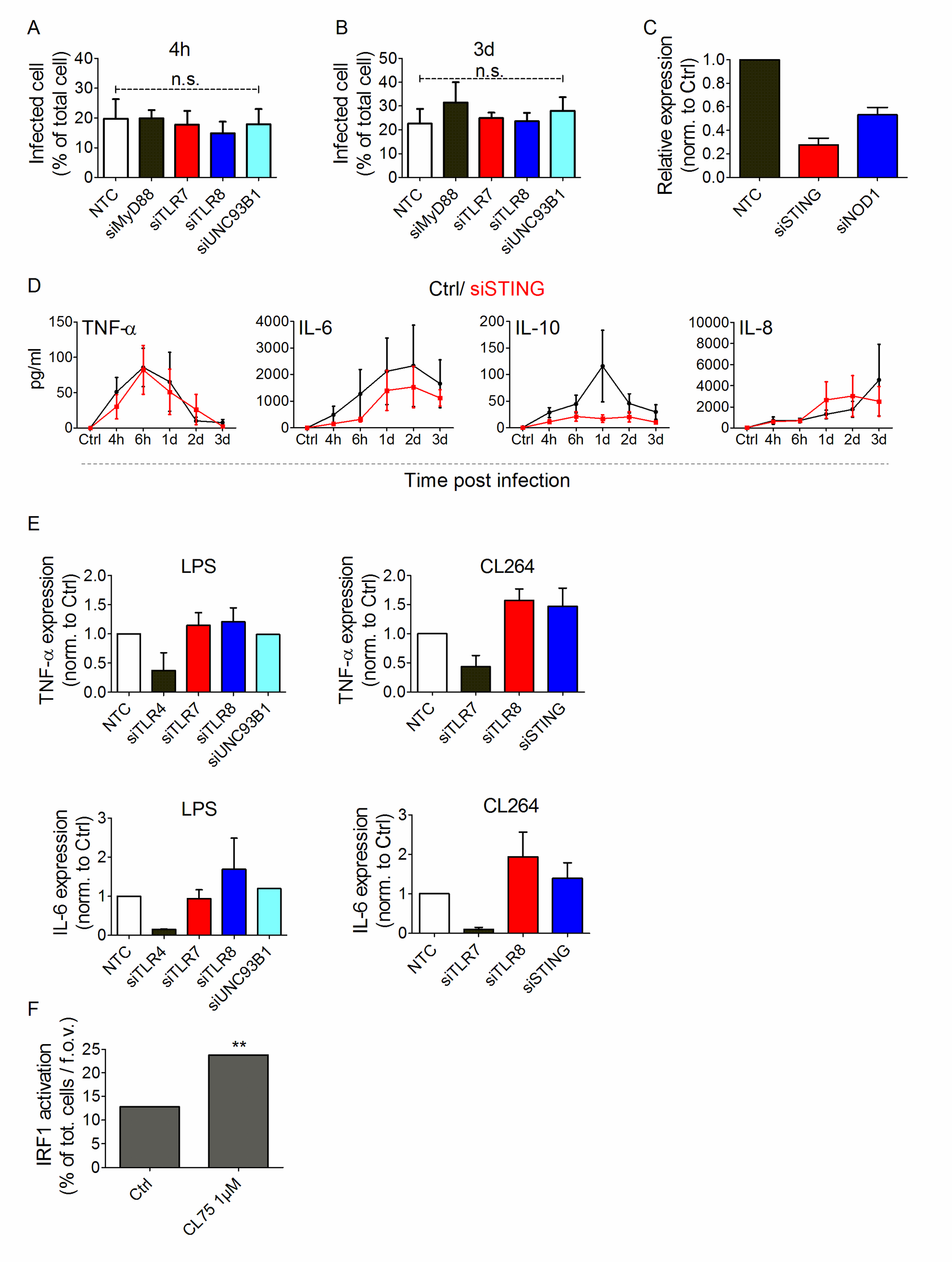

Supplement: S8 Fig — (A, B) Human MDMs were treated with siRNA against MyD88, TLR7, TLR8 or UNC93B1 and infected with Mav-CFP (10 min exposure followed by chase for 4h or 3d), fixed with PFA 4% and analyzed using confocal microscopy. Percentages of infected cells (mean +/- SEM) at 4 hours compare Mav uptake (A) and Mav cell-to-cell spread at 3 days post infection (B). (C, D) Human MDMs were treated with siRNA against STING or NOD1 and infected with Mav-CFP (10 min exposure followed by chase for 4h or 3d). (C) Knockdown efficiency for siSTING and siNOD1. Genes of interest are shown as mRNA fold of induction relative to control and normalized to GAPDH. (D) Cell supernatants were harvested at the indicated time points post infection and cytokine responses from siSTING treated MDMs assessed by multiplex ELISA. Graphs represent average concentrations +/- SEM of TNF-α, IL-6, IL-10 and IL-8 from 4 donors treated with siSTING (red) or non-targeted siRNA (black). (E) siRNA treated cells were challenged with 25 μg/ml LPS or 2.5 μM of the TLR7 ligand CL264 for 4 hours and relative TNF-α and IL-6 expression assessed by RT-qPCR. (F) Human MDMs were treated with 1 μM of the TLR8 ligand CL75 for 4 hours, stained with anti-IRF1 antibodies together with a nuclear stain (Hoechst) and analyzed for nuclear translocation of IRF-1 using confocal microscopy. Quantification graphs represent the mean value (n>700 cells per time point) of one representative donor from three independent experiments. P values were calculated using Fisher Exact test (* <0.05, ** <0.01 and *** <0.005.) (TIF) [file ppat.1006551.s008.tif]
